# A ribbon graph derivation of the algebra of functional renormalization for random multi-matrices with multi-trace interactions

**DOI:** 10.1007/s11005-022-01546-x

**Published:** 2022-06-11

**Authors:** Carlos I. Pérez-Sánchez

**Affiliations:** 1 Institute for Theoretical Physics, University of Heidelberg, Philosophenweg 19, 69120 Heidelberg, Germany; 2 Faculty of Physics, University of Warsaw, ul. Pasteura 5, 02-093 Warsaw, Poland

**Keywords:** Functional renormalization, Random matrices, Non-commutative algebra, Multi-matrix models, Ribbon graphs, 81-XX, 15B52, 05Cxx, 08B20, 46L54, 60B20

## Abstract

We focus on functional renormalization for ensembles of several (say $n\ge 1$) random matrices, whose potentials include multi-traces, to wit, the probability measure contains factors of the form $\exp [-\mathrm {Tr}(V_1)\times \cdots \times \mathrm {Tr}(V_k)]$ for certain noncommutative polynomials $V_1,\ldots ,V_k\in {\mathbb {C}}_{\langle n \rangle }$ in the *n* matrices. This article shows how the “algebra of functional renormalization”—that is, the structure that makes the renormalization flow equation computable—is derived from ribbon graphs, only by requiring the one-loop structure that such equation (due to Wetterich) is expected to have. Whenever it is possible to compute the renormalization flow in terms of $\mathrm U(N)$-invariants, the structure gained is the matrix algebra $M_n( \mathcal {A}_{n,N}, \star )$ with entries in $\mathcal {A}_{n,N}=({\mathbb {C}}_{\langle n \rangle } \otimes {\mathbb {C}}_{\langle n \rangle } )\oplus ( {\mathbb {C}}_{\langle n \rangle } \boxtimes {\mathbb {C}}_{\langle n \rangle })$, being ${\mathbb {C}}_{\langle n \rangle }$ the free algebra generated by the *n* Hermitian matrices of size *N* (the flowing random variables) with multiplication of homogeneous elements in $\mathcal {A}_{n,N}$ given, for each $P,Q,U,W\in {\mathbb {C}}_{\langle n \rangle }$, by

$$\begin{aligned} (U \otimes W) \star ( P\otimes Q)&= PU \otimes WQ \,, \\ (U\boxtimes W) \star ( P\otimes Q)&=U \boxtimes PWQ \,, \\ (U \otimes W) \star ( P\boxtimes Q)&= WPU \boxtimes Q \,, \\ (U\boxtimes W) \star ( P\boxtimes Q)&= \mathrm {Tr} (WP) U\boxtimes Q \,, \end{aligned}$$

which, together with the condition $(\lambda U) \boxtimes W = U\boxtimes (\lambda W)$ for each complex $\lambda$, fully define the symbol $\boxtimes$.

## Introduction and motivation

By the *Functional Renormalization Group* (FRG), physicists refer to a certain flow in the renormalization time *t*, usually the logarithm $t=\log k$ of the energy scale *k*, which in the “nonperturbative” [1] setting is governed by Wetterich equation [2]

$$\begin{aligned} \text {``}\partial _t \Gamma _{k}[\phi ]= \frac{1}{2} \mathrm {STr}\bigg (\frac{\partial _t R_{k} }{ {{\,\mathrm{Hess}\,}}\Gamma _{k}[\phi ]+R _{k}}\bigg )\text {''}\,. \end{aligned}$$

This is satisfied by the “effective action $\Gamma _k[\phi ]$, infrared-regulated by $R_k$ up to the energy scale *k*, on some space of fields $\phi$” (quotation marks, since mathematical details follow for the system of our interest). This article addresses functional renormalization for ensembles of *n*-tuples of Hermitian matrices; the particular type of ensembles we analyze have clear physical motivations (Sects. 1.1 and 1.2).

While there is no better way to compute it, the denominator in the right-hand side of Wetterich equation is a Neumann expansion (geometric series) in “${{\,\mathrm{Hess}\,}}\Gamma _k [\phi ] /R_k$”, essentially, the Hessian of the fields. For matrix ensembles, this Hessian is an object of four indices, two from each of the two derivatives. The question is which is the meaning of the product $\star$ implied in powers $({{\,\mathrm{Hess}\,}}\Gamma _k[\phi ])^{\star m}$ of the Hessian; we call the algebra defined by such product the *algebra of functional renormalization*.^1^

Of course, this question can be answered directly by looking at the proof of Wetterich equation; for the field theory in question, see [3]. For multi-matrix ensembles with probability measures defined, as is usual, solely in terms of single traces of matrix polynomials, part of the answer relies on observing that the Hessian is spanned, as $\sum _\alpha F_\alpha \otimes G_\alpha$, by couples of noncommutative polynomials $F_\alpha ,G_\alpha$. The (so far, unsurprising) answer is that powers of the Hessian are obtained by the product rule

$$\begin{aligned} ( U\otimes W)\star (P \otimes Q) = PU \otimes WQ \,. \end{aligned}$$

**Fig. 1:**
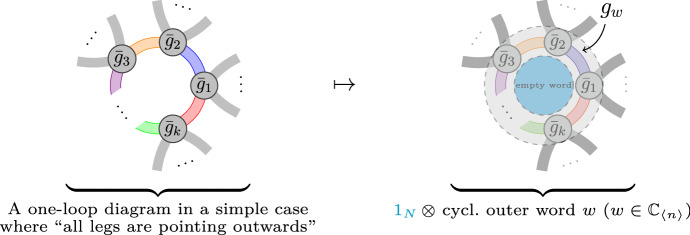
The colored legs correspond to $$ M_n$$-block entries  of the effective action $$\Gamma $$. Left: Unrenormalized interactions $${\bar{g}}_i$$ appearing in a *k*th power of the Hessian. Right: The contribution to the $$\beta _w$$-function, *w* formed by reading off clockwise the legs

**Fig. 2:**
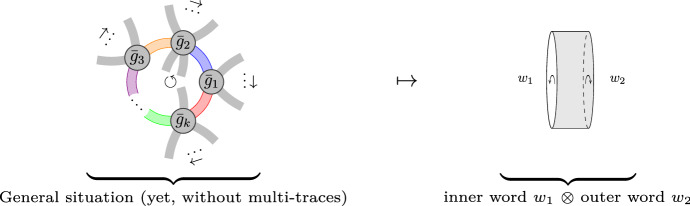
How the one-loop structure of the FRG is encoded in $M_n(\mathcal {A}_n,\star )$. *Left:* Unrenormalized interactions ${\bar{g}}_i$ appearing in a *k*th power of the Hessian. *Right:* Unlike Fig. 1, this situation leads to a cylindric topology. Each word $w_1$ and $w_2$ distributed at the boundary is oriented in a consistent way with an orientation of the cylindric surface (determined by the cyclic clockwise order in the interaction vertices)

Notice the “inversion” in one of the first tensor-factors, which starts to reflect the inner boundary and the outer one of the one-loop, relevant in this note (see Figs. 1 and 2). Interestingly, the incorporation of double traces yields a less trivial answer, for a “second product” appears (if one wants, a twisted tensor product) that also satisfies bilinearity $( z P) \boxtimes Q = P \boxtimes (zQ )$, $z\in {\mathbb {C}}$, but which differs from the usual tensor product only in the way one multiplies it with another element, $U\otimes W$ or $T\boxtimes V$. From interactions of two (or more) traces, then the Hessian of the effective action turns out to be spanned by noncommutative polynomials in a more general position^2^$\sum _{\alpha } F_\alpha \otimes G_\alpha + \sum _\rho H_\rho \boxtimes I _\rho$. The product reads

1a $$\begin{aligned} (U \otimes W+ Y\boxtimes Z) \star ( P\otimes Q)&= PU \otimes WQ + Y \boxtimes PZQ \,, \end{aligned}$$

1b $$\begin{aligned} (U \otimes W+Y\boxtimes Z) \star ( T\boxtimes V)&= WTU \boxtimes V+ {{\,\mathrm{Tr}\,}}_{N}(WT) U\boxtimes V\,. \end{aligned}$$

The aim of this article is to prove, using graphs, that the sole assumption that the contributions to the rhs of Wetterich equation have *all* a “one-loop structure” implies that the rhs of Wetterich equation satisfies Eq. (1). Next, we justify the appearance of noncommutative (nc) polynomials and of double traces, relating both with other theories (in Sects. 1.1 and 1.2, respectively). In Sect. 2, before presenting the precise statement, we give a short, but self-contained account of the ribbon graph theory needed to prove, in Sect. 3, the main statement (Theorem 2). “Appendix A” explains the construction of the infrared-regulated effective action.

### The origin of the noncommutative polynomials and potential applications

Ensembles of several matrices with probability laws given by ordinary (commutative) potentials are extensively studied in high-energy physics. An important family of models solved by Eynard–Orantin [4], using their topological recursion, is the *two-matrix model*, which refers to measures $\mathrm {d}\mu$ on $\mathcal {H}_N^2$ of the form:

2 $$\begin{aligned} \mathrm {d}\mu (A,B)&= \exp [-{{\,\mathrm{Tr}\,}}_{N}(AB)] \nonumber \\&\quad \times \exp \big \{-{{\,\mathrm{Tr}\,}}_{N}[V_1 (A)\big \} (\mathrm {d}A){}_{\textsc {Leb}}\times \exp \big \{-{{\,\mathrm{Tr}\,}}_{N}[V_2 (B)]\big \} (\mathrm {d}B){}_{\textsc {Leb}}\,. \end{aligned}$$

Modulo the first factor, this is still a product of measures, each of which on the space $$\mathcal {H}_N$$ of $$N\times N$$ Hermitian matrices. Here, $$V_1(x) $$ and $$V_2(x)$$ are polynomials in a real variable *x* and $${{\,\mathrm{Tr}\,}}_{N}(X)=\sum _{i=1}^N X_{i,i}$$ is the unnormalized trace. We will keep this notation in the sequel.

The simplest addressed and (using the character expansion method [5]) solved model with a genuinely noncommutative law, generalizing Eq. (2), is the *ABAB*-model with measure

3 $$\begin{aligned} \mathrm {d}\mu (A,B)&= \exp \big \{-N {{\,\mathrm{Tr}\,}}_{N}(g_{A^4} A^4+ g_{B^4} B^4 + g_{ABAB} ABAB) \big \} \mathrm {d}\gamma (A, B) \nonumber \\&=:\exp (-S{}^{\textsc {Int}}[A,B])\mathrm {d}\gamma (A, B), \end{aligned}$$

where

4 $$\begin{aligned} \mathrm {d}\gamma (A,B)= \exp \Big \{-\frac{N}{2}{{\,\mathrm{Tr}\,}}_{N}(A^2+B^2)\Big \} (\mathrm {d}A){}_{\textsc {Leb}}(\mathrm {d}B){}_{\textsc {Leb}}\end{aligned}$$

is the product Gaussian measure on $\mathcal {H}_N^2$. The action *S* that defines the probability measure $\mathrm {d}\mu =\exp (-S[A,B])(\mathrm {d}A){}_{\textsc {Leb}}(\mathrm {d}B){}_{\textsc {Leb}}$ is the *bare action*. Hermitian ensembles with wildly non-factorizable measures, as those relevant in this paper, generalizing (3), are studied in free probability [6].

A more recent application of nc polynomial interactions concerns ensembles of Dirac operators

5 $$\begin{aligned} {\mathcal {Z}}=\int _{\mathrm {Dirac}} \exp [-S(D) ] \mathrm {d}D \end{aligned}$$

which aim at the quantization of the spectral action $S(D)={{\,\mathrm{Tr}\,}}f(D)$ in noncommutative geometry. This problem was posed since [7, Ch. 19] and finite approximations to smooth geometries that allow to make precise sense of the partition function (5) recently reawakened interest in the problem [8–11]. In the spectral action [12, 13], traces of *f*(*D*) yield (nontrivially noncommutative) polynomial interactions as well as double traces, cf. Eq. (6).

Applications of nc polynomial interactions were relevant for a better understanding of the Temperley–Lieb algebra.^3^ From a Temperley–Lieb vertex ${\mathcal {B}}$, i.e., a rooted, planar chord diagram one obtains a nc polynomial as in the next example:Nc polynomial matrix interactions are also auxiliary in the description of more general planar algebras [14] and $O({\mathfrak {n}})$-loop models.

### On multi-trace interactions

We will see later that not including multi-trace interactions in renormalization is unnatural (since generic radiative corrections include more traces than the bare action did). This short section mentions theories that contain multi-traces even before addressing renormalization (whenever possible).

**Fig. 3:**
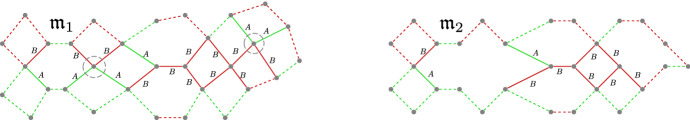
Example of *(face-)worded maps* ${\mathfrak {m}}_1$ and ${\mathfrak {m}}_2$ dual to ribbon graphs generated by multi-matrix models with noncommutative polynomial interactions. Each *r*-agon of sides marked with letters $X_{i_1}\ldots X_{i_r}$ is generated by the interaction vertex ${{\,\mathrm{Tr}\,}}_{N}(X_{i_1}\ldots X_{i_r} )$. The relation between ${\mathfrak {m}}_1$ and ${\mathfrak {m}}_2$ is the renormalization flow. In the cross graining process, the one-loop configurations at the nodes marked with dashed circles in ${\mathfrak {m}}_1$ yield the effective (in this case, higher-degree) interactions in ${\mathfrak {m}}_2$. This is the dual version of the cross graining depicted in Fig. 1 (Dashed edges mean that the maps can extend in that direction and might get some non-planar topology)

- *Dirac ensembles* always yield double trace interactions (then renormalization creates even more traces). The (bare) Dirac ensemble measure is of the type
  6 $$\begin{aligned} \mathrm {d}\mu (X_1,\ldots , X_n )= \exp \big \{ -N {{\,\mathrm{Tr}\,}}_{N}(P) - {{\,\mathrm{Tr}\,}}_{N}^{\otimes 2 } (Q_{(1)}\otimes Q_{(2)} ) \big \} \mathrm {d}(X_1,\ldots , X_n ){}_{\textsc {Leb}}\end{aligned}$$
  for *P*, $Q_{(1)}$ and $Q_{(2)}$ also^4^ nc polynomials in the matrices $X_1,\ldots , X_n$ and $\mathrm {d}(X_1,\ldots , X_n ){}_{\textsc {Leb}}$ is the product Lebesgue measure, now on $\mathcal {H}_N^n$. Even though *P* has an extra factor of *N* with respect to the double trace, observe that the latter cannot be neglected, since ${{\,\mathrm{Tr}\,}}_{N}(Q_{(1)}) \times {{\,\mathrm{Tr}\,}}_{N}(Q_{(2)})$ contains a double sum too.
- *Face-worded, stuffed maps.* Combinatorial maps (“gluing of polygons” dual to ribbon graphs) are counted with the aid of matrix partition functions [15]. In the presence of two random matrices with the probability law (2), the faces of these maps can be uniformly colored (and interpreted as Ising model) [16, §8]. If the potentials are noncommutative polynomials, this is no longer possible, and the partition function generates maps whose faces are labelled by “cyclic words” in the matrices (thus the maps could be called *face-worded*, as presented in Fig. 3 for the alphabet $\{A,B\}$). If the interaction vertices have several traces, the generated maps are said to be *stuffed* [11, 17] (independent of whether the potentials are ordinary or noncommutative). The terminology reflects that one now allows maps to have elementary cells of a topology that need not be that of a disk, i.e., one has “maps stuffed with bordered Riemann surfaces”. The renormalization flow we study yields equations for the $\beta$-functions for matrix ensembles whose partition function generate “face-worded, stuffed maps”. The fixed-point solution of the $\beta$-function system Eq. (14) could be useful to compute critical exponents (see Remark 1).
- *“Touching interactions”*. In several quantum gravity approaches, multi-trace operators appear, to name only few:
  - in *Liouville gravity*, multi-trace one-matrix models are interpreted as generating functions of surfaces that might touch at isolated points. (The planar sector, for instance, is grasped, according to [18], as trees of spheres that can touch other spheres at most once.)
  - multi-trace interactions appear in *curvature matrix models* [19]. Double traces appear in the effective description of a matrix model with a kinetic term ${{\,\mathrm{Tr}\,}}(\phi E\phi E)$ (with broken symmetry by a constant matrix *E*).
  - another interpretation in terms of wormholes appears in (a certain two-matrix model description of) 3-dimensional *Causal Dynamical Triangulations* [20]
  - under the *AdS/CFT*-correspondence, the AdS-object matching multi-trace operators in CFT are multi-particle states. In this context, for those states [21] defines the natural boundary conditions at $\infty$.

## Terminology and main statement

Since our aim is to connect combinatorics and algebra in matrix models, on the one hand, with renormalization on the other, this article is somewhat interdisciplinary. Therefore, it is convenient to precisely define our framework and notation.

### Ribbon graphs and the noncommutative Hessian on single trace interactions

The next points introduce our notation and present some definitions:

- The space of Hermitian $N\times N$ matrices is denoted by $\mathcal {H}_N$. The size of matrices $X_1{}^{(N)},X_2{}^{(N)},\ldots , X_n{}^{(N)}\in \mathcal {H}_N$ (which will become the random variables) will be relevant, but the lighter notation $X_1,X_2, \ldots , X_n$ is convenient. The number *n* of matrices remains fixed, and we will denote the *n*-tuple $(X_1,X_2,\ldots , X_n )$ by ${\mathbb {X}}$.
- ${\mathbb {C}}_{\langle n\rangle }={\mathbb {C}}\langle X_1,X_2,\ldots , X_n \rangle$ is the *free algebra*. Any element of ${\mathbb {C}}_{\langle n\rangle }$ is spanned by words in the alphabet ${\mathbb {X}}$, and ${\mathbb {C}}_{\langle n\rangle }$ is endowed with the concatenation product. We actually should write ${\mathbb {C}}_{\langle n\rangle , N}$ instead of ${\mathbb {C}}_{\langle n\rangle }$ emphasizing that the generators $X_a$ are matrices of size *N*, but the only manifestation of it is the empty word being the unit matrix $1_N$, and we opt again for a light notation.
- The *noncommutative derivative* with respect to *A*, $\partial _{A} :{\mathbb {C}}_{\langle n\rangle } \rightarrow {\mathbb {C}}_{\langle n\rangle }^{\,\otimes \,2}$ on a word *w* containing *A* is the sum over “replacements of *A* in *w* by the $\otimes$ tensor product symbol” in the middle of the word; if *A* occurs at the left (resp. right) end, then one additionally attaches the empty word (or in ${\mathbb {C}}_{\langle n\rangle , N}$ a unit $1_N$) to the left (resp. right) of $\otimes$. For example, in a free algebra with enough letters
- The noncommutative derivative defined on “cyclic words” ${{\,\mathrm{Tr}\,}}P$, $P\in {\mathbb {C}}_{\langle n\rangle }$, is given by the sum of all possible excisions $P\setminus A$ of *A* from *P*, rooting (i.e., starting) the remaining word at the letter after the removed *A*
  $$\begin{aligned} \partial _A: {\text {im}}{{\,\mathrm{Tr}\,}}\rightarrow {\mathbb {C}}_{\langle n\rangle }, \quad {{\,\mathrm{Tr}\,}}P \mapsto \sum _{\begin{array}{c} \text {rootings at}\\ A\text {'s next letter} \end{array}} P \setminus A\,. \end{aligned}$$
  The result $\partial _A {{\,\mathrm{Tr}\,}}P=: {\mathscr {D}}_A P$ defines the *cyclic derivative* ${\mathscr {D}}_A$ of *P* and is due to Rota-Sagan-Stein [22] and Voiculescu [23]. For instance, . The adjective “cyclic” for ${\mathscr {D}}$ comes from the property ${{\,\mathrm{{\mathscr {D}}}\,}}_{X_a} P = {{\,\mathrm{{\mathscr {D}}}\,}}_{X _a}[\sigma (P)]$, which holds for any cyclic permutation $\sigma (P)$ of the letters of *P* ($P\in {\mathbb {C}}_{\langle n\rangle }$ and any $a=1\ldots ,n$).
- Grasping ${{\,\mathrm{Tr}\,}}$ as the trace in ${\mathbb {C}}_{\langle n\rangle }$ induced by that of $M_N({\mathbb {C}})$, define the *noncommutative Hessian* [3] of a cyclic word
  7 $$\begin{aligned} \quad {{\,\mathrm{Hess}\,}}: {\text {im}}{{\,\mathrm{Tr}\,}}&\rightarrow M_n( {\mathbb {C}}_{\langle n\rangle }\otimes {\mathbb {C}}_{\langle n\rangle }) \nonumber \\ {{\,\mathrm{Tr}\,}}P&\mapsto ({{\,\mathrm{Hess}\,}}_{a,b} {{\,\mathrm{Tr}\,}}P)_{a,b=1,\ldots ,n}:=(\partial _{X_a}\circ \partial _{X_b} {{\,\mathrm{Tr}\,}}P)_{a,b=1,\ldots ,n} \,. \end{aligned}$$
  Referring to the block $M_n$-matrix structure, i.e., to indices $a,b=1\ldots ,n$, notice that in general the nc Hessian is not a symmetric matrix, ${{\,\mathrm{Hess}\,}}_{a,b} {{\,\mathrm{Tr}\,}}P \ne {{\,\mathrm{Hess}\,}}_{b,a} {{\,\mathrm{Tr}\,}}P$.

The (*b*, *a*)-entry in the $M_n$-matrix block structure of the Hessian of a cyclic word ${{\,\mathrm{Tr}\,}}W$ can be represented graphically by summing over all the *ordered* double markings of $X_a$ and $X_b$ inside a word *W*. On $W= X_{\ell _1}X_{\ell _2} \cdots X_{\ell _{k}}\in {\mathbb {C}}_{\langle n\rangle , N}$ (with $k\ge 2$), according to Eq. (7), this is given for $a,b=1,\ldots ,n$ by (for a proof see [3, Prop. 2.3])8We sum over all oriented pairings $\pi =(uv)$ between the letters of the cyclic word ${{\,\mathrm{Tr}\,}}W$ (which explains the circle in the second equality). In Eq. (8), $\pi _1(W)$ is the ordinary word between $X_{\ell _u}$ and $X_{\ell _v}$ and $\pi _2(W)$ that between $X_{\ell _v}$ and $X_{\ell _u}$, and because of the deltas $X_{\ell _v} =X_b$ and $X_{\ell _u} =X_a$ must hold, and the empty word in either case leads to writing $1_N$.

#### Example 1

To simplify the drawings, we expose the case $n=2$. We compute the nc Hessian entry corresponding to $X_a=A$ and $X_b=B$ on ${{\,\mathrm{Tr}\,}}_{N}W= {{\,\mathrm{Tr}\,}}_{N}(ABAABABB)$. The entry reads:The cyclicity is lost due to each cut (dashed line). The word represented by each excision is read starting from the letter right after^5^ the cut: the first one is $AABABB, \ldots$ , and the sixth *BBABAA*. These terms that are listed arise from contiguous appearances of *AB* and *BA* in *W* and in each case the empty word between the letters originates the $1_N$ tensor factor. According to Eq. (8), the rest of the polynomials (last line) are computed by cutting the circle into two non-trivial words. For instance, the next cut yields9The order of the derivatives $\partial _B \partial _A$ (to the left of the cut “from *A* to *B*”, $\Rightarrow$ first factor, to the left of “from *B* to *A*” $\Rightarrow$ second factor) determines which word is placed in which tensor factor.

### Multi-trace interaction vertices, effective vertices

The interaction vertices in the measures $\mathrm {d}\mu ({\mathbb {X}})= \exp \big \{ -N {{\,\mathrm{Tr}\,}}_{N}(P) - {{\,\mathrm{Tr}\,}}_{N}^{\otimes 2 } (Q_{(1)}\otimes Q_{(2)} ) \big \} \mathrm {d}\gamma ({\mathbb {X}})$ are represented by ribbon vertices framed with a dashed circle. This is unusual, but in view of the multiple products of traces in the measure, a helpful notation. The coupling constant ${\bar{g}}$ of multiple trace interactions is what prevents the multiple traces from being interpreted as different, disconnected polygonal building blocks (and are interpreted as “touching-interactions” [18–21] in other settings). Their relation to the free algebra is explained with the following examples (where green/light means the *A* matrix and red/dark represents *B*)101112The convention is that the label of the coupling constants applies to everything inside the dashed circle, i.e., simultaneously both traces (see also Example 5). This representation also reflects the mathematical nature of the effective action $\Gamma _N[{\mathbb {X}}]$ as (for now, at least) a formal series (with the coupling constants as parameters) of the form:

13 $$\begin{aligned} \Gamma _N[{\mathbb {X}}]&= \sum _\alpha O_\alpha \qquad O_\alpha = {\bar{g}}_{\alpha } \prod _{r=1}^{t_\alpha } {{\,\mathrm{Tr}\,}}_N(w_{\alpha ,r})\,,\quad \nonumber \\&\text { for certain monomials }w_{\alpha ,r} \in {\mathbb {C}}_{\langle n\rangle }= {\mathbb {C}}\langle {\mathbb {X}} \rangle \end{aligned}$$

so $t_\alpha$ is the number of traces in the *operators* $O_\alpha$. The monomials $w_{\alpha ,r}$ need not be monic; as a matter of fact, one usually normalizes $w_{\alpha ,r}$ with symmetry factors. The coefficient of the kinetic operator ${{\,\mathrm{Tr}\,}}( X_c^2/2)$ (for each $c=1,\ldots ,n$) is called the wave function renormalization (of the matrix $X_c$) and, since it is special, it is usually denoted not by a ${\bar{g}}$ but by $Z_c$. Else, we call *interaction vertices* the remaining $O_\alpha$’s. The bar on the coupling constant ${\bar{g}}_i={\bar{g}}_i(N)$, which are functions of *N*, denotes that it will still be rescaled ${\bar{g}}_\alpha \rightarrow g_\alpha =Z^{\lambda _\alpha } N^{\kappa _\alpha } {\bar{g}}_\alpha$, solving for $\lambda _\alpha$ and $\kappa _\alpha$, in order to render finite and *Z*-independent the next system (only in the large-*N* differential^6^) equations

14a $$\begin{aligned} \big \{\eta _c:\!&=-\partial _t \log Z_c = -Z_c{}^{-1}\times \text {coeff. of } {{\,\mathrm{Tr}\,}}_N(X_c^2/2) \text { in rhs of Eq.} (.28) \big \}_{c=1,\ldots ,n} \end{aligned}$$

14b $$\begin{aligned} \Big \{ \beta _\alpha :\!&= \partial _t g_\alpha = \text {coeff. of }\prod _{r=1}^{t_\alpha } {{\,\mathrm{Tr}\,}}_N(w_{\alpha ,r}) \text { in the rhs of Eq.} (28) \Big \}_{\alpha },\, t=\log N\, \end{aligned}$$

of ($\eta$-functions and) $\beta$-function equations for the interaction vertices $\alpha$, determined by Wetterich equation. This list of operators appearing in Eq. (14) includes those of the original (bare) action *S*, but additionally those generated from it by “radiative corrections” to *S*. For instance^7^ if the initial model is given by

15 $$\begin{aligned} S=N {{\,\mathrm{Tr}\,}}_{N}\Big \{ \frac{1}{2} A^2+\frac{1}{2} B^2 + g_{A^4}\frac{1 }{4}A^4+ g_{B^4}\frac{1}{4} B^4 +\frac{1}{2} g_{ABAB} ABAB \Big \} \end{aligned}$$

then the radiative corrections16“generate” the *effective vertex* $N {{\,\mathrm{Tr}\,}}_{N}(ABBA)$ (see below, how). Also disconnected vertices are generated; for instance, ${{\,\mathrm{Tr}\,}}_{N}(A)\times {{\,\mathrm{Tr}\,}}(A)$ is generated from $A^4$ (by contracting non-consecutive half-edges) and from *ABAB* (by contracting the *B* edges).

Therefore, the effective action should include these (and all corrections), and becomes^8^

17 $$\begin{aligned} \Gamma _N[A,B]&= {{\,\mathrm{Tr}\,}}_{N}\left\{ \overbrace{\frac{Z_A}{2} A^2+\frac{Z_B}{2} B^2 + {\bar{g}}_{A^4}\frac{1 }{4}A^4+ {\bar{g}}_{B^4}\frac{1}{4} B^4+\frac{1}{2} {\bar{g}}_{ABAB} ABAB }^{\text {operators from the bare action (but with ``running couplings'')}}\right. \nonumber \\&\quad \,\,\,\, \left. + \underbrace{\frac{1}{2}{\bar{g}}_{ABBA} ABBA + \frac{1}{2}{\bar{g}}_{A \mid A}{{\,\mathrm{Tr}\,}}_{N}(A)\times A +\cdots }_{\text {radiative corrections}} \right\} \end{aligned}$$

The effective vertices are obtained by taking the boundary graph of the radiative corrections. In other words, they are constructed from a Feynman graph—as were those in (16) for the model (15)—as defined next, and explained with examples immediately thereafter.

#### Definition 1

(Effective interaction vertex) Given a Feynman graph *G* of a multi-trace multi-matrix model, first single out the traces $${{\,\mathrm{Tr}\,}}_{N}(U_1),\ldots ,{{\,\mathrm{Tr}\,}}_{N}(U_r)$$ that are not contracted by a propagator. Second, pick an arbitrary side of a ribbon-propagator and travel along the diagram with the orientation induced by the clockwise orientation of the interaction vertices, listing in that order the letters that label the half-edges of these (cf. Example 3) until one comes back to the initial, chosen propagator (on the same side); call the thus obtained word $$ w_1$$. Repeat this process picking an unvisited side of a propagator, and iterate until all ribbon propagators visited once by both sides (and thus all uncontracted half-edges are listed exactly once), say, at the *s*th iteration. The effective vertex $$O_G^{\mathrm {eff}}$$ of the graph *G* is defined by

$$\begin{aligned} O_G^{\mathrm {eff}}=\underbrace{{{\,\mathrm{Tr}\,}}_{N}(w_1)\times {{\,\mathrm{Tr}\,}}_{N}(w_2)\times \cdots \times {{\,\mathrm{Tr}\,}}_{N}(w_s)}_{\text {from vertices contracted with propagators}} \times \!\!\underbrace{ {{\,\mathrm{Tr}\,}}_{N}(U_1)\times {{\,\mathrm{Tr}\,}}_{N}(U_2)\cdots \times {{\,\mathrm{Tr}\,}}_{N}(U_r)}_{\text {from vertices uncontracted with propagators}} \end{aligned}$$

Since the words appear inside the trace, the construction is evidently independent of the propagators we started with to construct each word $w_1,\ldots ,w_s$.

#### Example 2

(Graphs containing an empty loop.) To illustrate the effective vertex construction of a two-matrix model, consider the graph on the right, which corresponds to a correction from the operators

18 $$\begin{aligned} O_1&= {\bar{g}}_1 {{\,\mathrm{Tr}\,}}_{N}(B^3ABA)\,, \end{aligned}$$

19 $$\begin{aligned} O_2&= {\bar{g}}_2 {{\,\mathrm{Tr}\,}}_{N}(A^2) {{\,\mathrm{Tr}\,}}_{N}(A^3BAB) \,. \end{aligned}$$

The effective vertex is $N {\bar{g}}_1{\bar{g}}_2 {{\,\mathrm{Tr}\,}}_{N}(A^2) {{\,\mathrm{Tr}\,}}_{N}(B^3A^2BA^2)$. The quadratic trace comes from the uncontracted trace in $O_2$; the long word comes from the “outward” loop and the factor $N={{\,\mathrm{Tr}\,}}_{N}1_N$ from the inner, empty word.


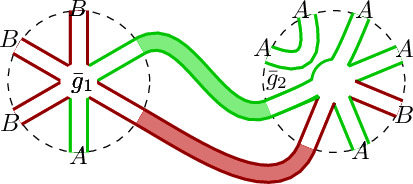


#### Example 3

(Orientation of loops.) With the operators

20 $$\begin{aligned} O_1&= {\bar{g}}_1 {{\,\mathrm{Tr}\,}}_{N}( CFBDEA)\,, \end{aligned}$$

21 $$\begin{aligned} O_2&= {\bar{g}}_2 {{\,\mathrm{Tr}\,}}_{N}( CDFABCEA) \,, \end{aligned}$$

we now illustrate the orientation of the loops. Each operator endows the interaction vertex with an orientation. The effective vertex should be read off respecting it. This means that outward loops are clockwise oriented and inward loops anti-clockwise. The effective vertex is ${\bar{g}}_1{\bar{g}}_2 {{\,\mathrm{Tr}\,}}_{N}( CFCE) \times {{\,\mathrm{Tr}\,}}_{N}(CDFADE )$.


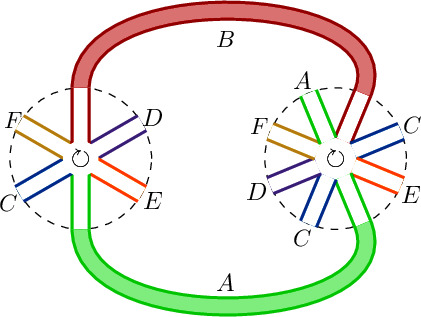


#### Example 4

(Propagators joining different traces in the same interaction vertex.) Consider now the graph on the right.

22 $$\begin{aligned} O_1&= {\bar{g}}_1 {{\,\mathrm{Tr}\,}}_{N}(ADBADB)\,, \end{aligned}$$

23 $$\begin{aligned} O_2&= {\bar{g}}_2 {{\,\mathrm{Tr}\,}}_{N}(BACDBACD)\,, \end{aligned}$$

24 $$\begin{aligned} O_3&= {\bar{g}}_3 {{\,\mathrm{Tr}\,}}_{N}(C^2)\times {{\,\mathrm{Tr}\,}}_{N}(D^3BDB) \,, \end{aligned}$$

25 $$\begin{aligned} O_4&= {\bar{g}}_4 {{\,\mathrm{Tr}\,}}_{N}(A^4) \times {{\,\mathrm{Tr}\,}}_{N}(D^6) \,, \end{aligned}$$

(and possibly other more operators making the action real). The effective vertex is ${\bar{g}}_1{\bar{g}}_2 {\bar{g}}_3 {\bar{g}}_4$ $\times$ ${{\,\mathrm{Tr}\,}}_{N}(BDBD^7){{\,\mathrm{Tr}\,}}_{N}(A^3DACDBACDADB)$. This graph is also a one-loop (see Definition 2 for the subtleties that appear in the presence of multi-trace interactions).


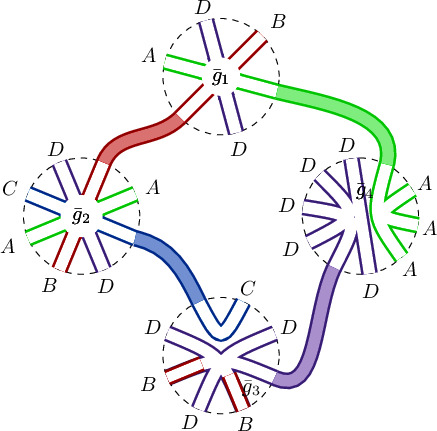


In the presence of multi-traces, the one-loop condition cannot be formulated purely in terms of the first Betti-number $b_1(G)$. Instead

#### Definition 2

Let *G* be a ribbon graph of a multi-trace multi-matrix model. We denote by $G^{\circ }$ the one-dimensional skeleton obtained after collapsing the interaction vertices^9^ to points and the propagators (edges between interaction vertices) to ordinary edges. A *one-loop graph* of a multi-matrix model with multi-traces is a ribbon graph *G* whose skeleton $G^\circ$ is one-particle irreducible (1PI; or, equivalently, a 2-edge connected graph) and which additionally has a first Betti-number $b_1(G^{\circ })= 1$.

#### Example 5

The next three diagrams are all one-loop graphs:(We omit the coupling constants ${\bar{g}}_i$ by now, since we care about topology in this example). First, $G_1$ has $b_1(G_1^\circ )=1$; next, although $b_1(G_2) \ne 1$, since thinning the edges and collapsing the stars (dashed circles) to points yields a circle, $G_2^\circ$ does have first Betti-number 1. The same argument holds for $G_3$. Having these graphs explained the subtleties of the multiple traces, we give now ordinary examples. Regardingonly the *tadpole* $G_4$ is a one-loop graph; although $b_1(G_5^\circ )=1$, $G_5$ is not 1PI. And $G_6$ is such that $G_6^\circ$ has two loops, $b_1(G_6^\circ )=2$, so neither $G_5$ nor $G_6$ satisfy Definition 2.

### Including multi-traces and the main result

We denote by $N_{\infty }\in {\mathbb {N}}$ the energy scale at which the bare action describes the system.^10^ The renormalization flow modifies then the probability measure used to compute observables as follows. The starting point of the flow is the bare action *S* (or the measure $\mathrm {d}\mu {}^{\textsc {uv}}_{N_{\infty }}$ defined by it)

26 $$\begin{aligned} S:\mathcal {H}_{N_{\infty }}^n\rightarrow {\mathbb {R}}\qquad \mathrm {d}\mu {}^{\textsc {uv}}_{N_{\infty }}=\exp \Big \{-S\big [{\mathbb {X}}{}^{(N_{\infty })}\big ]\Big \} \mathrm {d}{\mathbb {X}}{}_{\textsc {Leb}}{}^{(N_{\infty })} \,. \end{aligned}$$

The “$\textsc {{uv}}$” in the measure emphasizes that the action *S* that defines the probability measure $\mathrm {d}\mu {}^{\textsc {uv}}_{N}=\exp \big \{-S[{\mathbb {X}}]\big \}(\mathrm {d}\mathbb {X}) {}_{\textsc {Leb}}$ is the bare action. In order to flow toward a lower energy scale $N< N_{\infty }$, a regulator $R_N$ takes care of integrating the *higher modes* (i.e., the matrix entries $N<i,j\le N_{\infty }$ of each of the *n* matrices; see Appendix A). This smoothens the idea of step-by-step integration [25] of the $N+1$th momentum shell, in order to obtain from ensembles of matrix of size $N+1$, effective ensembles of $N\times N$ matrices. This idea was put forward in [26] for the one-matrix model in a quantum gravity context. Other renormalization theories based on Polchinski equation have been addressed in [27].

The system at that lower scale *N* is described by the effective action $\Gamma _N$ and by the respective measure $\mathrm {d}\mu {}^{\mathrm {eff}}_N$ at the scale *N*,

27 $$\begin{aligned} \Gamma _N : {\left\{ \begin{array}{ll} \mathcal {H}_{N}^n\!\!\!\!\!\!&{} \rightarrow {\mathbb {R}}, \\ {\mathbb {X}}{}^{(N)}\!\!\!\!\!\!&{}\mapsto \Gamma _N[{\mathbb {X}}{}^{(N)}] \end{array}\right. } \qquad \mathrm {d}\mu {}^{\mathrm {eff}}_N({\mathbb {X}}{}^{(N)})= \exp \big \{-\Gamma _N {[}{\mathbb {X}}{}^{(N)} ]\big \} \mathrm {d}{\mathbb {X}}{}_{\textsc {Leb}}{}^{(N)}\,. \end{aligned}$$

It can be proven—rigorously, at least in the sense of formal series—that the effective action satisfies Wetterich equation [3],

28 $$\begin{aligned} \partial _t \Gamma _N[{\mathbb {X}}]= \frac{1}{2} \mathrm {STr}\Big (\frac{\partial _t R_N}{ {{\,\mathrm{Hess}\,}}\Gamma _N [{\mathbb {X}}]+R_N}\Big )\,, \end{aligned}$$

but as pointed out in the introduction, this is not the approach we follow in this article. We rather assume that the renormalization flow is governed by an equation of the form (28) and let ribbon graph theory dictate us the several objects that appear, specially the algebra obeyed by the Hessian. If an expansion in $\mathrm U(N)$-invariant operators exist, one is able to split the supertrace as follows:

29 $$\begin{aligned} \frac{1}{2}\mathrm {STr}\Big \{\frac{\partial _t R_N}{ {{\,\mathrm{Hess}\,}}\Gamma _N [{\mathbb {X}}]+R_N}\Big \}&= \sum _{k=0}^\infty \overbrace{ {\bar{h}}_k(N,\eta _1,\ldots ,\eta _n )}^{R_N\text {-dependent part}} \nonumber \\&\quad \times \underbrace{ \frac{1}{2}(-1)^k \mathrm {STr}\big \{ ({{\,\mathrm{Hess}\,}}\Gamma {}^{\textsc {Int}}_N [{\mathbb {X}}])^{\star k} \big \}}_{\text {regulator-independent part}}\,, \end{aligned}$$

where ${\bar{h}}_k(N, \{\eta _1,\ldots ,\eta _n\})$ is a function of *N* and the anomalous dimensions $\eta _c=-\partial _ t \log Z_c$; finally, $\Gamma {}^{\textsc {Int}}_N[{\mathbb {X}}]$ is the interaction part of $\Gamma _N$, which will be constructed below. Since we are looking for a “universal” algebras (not in the usual sense, but in the sense that they will appear independent on the regulator $R_N$), details on $R_N$ are placed in “Appendix A’.

In order to find the algebra ${\mathscr {A}}$ where the Hessian of the effective action lies, let us search for the identity element of ${\mathscr {A}}$. Because this algebra should contain $M_n({\mathbb {C}}_{\langle n\rangle }\otimes {\mathbb {C}}_{\langle n\rangle })$ (still seen as a vector space), we assume that ${\mathscr {A}}$ is also a matrix algebra of the form ${\mathscr {A}}=M_n(\mathcal {A}_n)$ for certain $\mathcal {A}_n$, and define the *supertrace*^11^$\mathrm {STr}$ on a matrix ${\mathcal {P}}=(P_{a,b})_{a,b=1,\ldots , n} \in M_n(\mathcal {A}_n)$, $P_{a,b} \in \mathcal {A}_n$ by

30 $$\begin{aligned} \mathrm {STr}({\mathcal {P}}) =\sum _{a=1} ^n {{\,\mathrm{Tr}\,}}_{\mathcal {A}_n}(P_{a,a})\, \end{aligned}$$

in terms of ${{\,\mathrm{Tr}\,}}_{\mathcal {A}_n}$, where $\mathcal {A}_n$, its product $\star$ and its trace ${{\,\mathrm{Tr}\,}}_{\mathcal {A}_n}$ are to be determined.

For this purpose, we observe that the effect of the kinetic terms, at a graph level, is just elongating the ribbons, and since all $R_N$-dependence has been absorbed in the coefficients ${\bar{h}}_k$ in Eq. (29), we conclude that the Hessian of the kinetic terms cannot modify the effective vertex at all: since, for $a,b,c,d\in \{1,\ldots ,n\}$, 31a31b for any interaction vertex *O*. On the other hand, the double trace terms $[{{\,\mathrm{Tr}\,}}X_c]^2$ “cut” the interaction vertex: 32a32b By Eq. (31), ${{\,\mathrm{Hess}\,}}_{c,c} \frac{1}{2} {{\,\mathrm{Tr}\,}}(X_c^2) = 1_N\otimes 1_N$ (no sum) is the left and right identity of $\mathcal {A}_{n,N}$, and by Eq. (32) there is another constant generator in $\mathcal {A}_{n,N}$ that, by the previous graph argument, is not proportional to $1_N\otimes 1_N$ (and therefore cannot be the identity) and which we denote by $1_N\boxtimes 1_N$.

#### Definition 3

We define $\mathcal {A}_n:={\mathbb {C}}_{\langle n\rangle }^{\,\otimes \, 2} \oplus {\mathbb {C}}_{\langle n\rangle }^{\,\boxtimes \,2}= [{\mathbb {C}}_{\langle n\rangle }\otimes {\mathbb {C}}_{\langle n\rangle }] \oplus [{\mathbb {C}}_{\langle n\rangle }\boxtimes {\mathbb {C}}_{\langle n\rangle }]$. Again, this is simplified notation for $\mathcal {A}_{n,N}$ defined as $\mathcal {A}_n$, but with ${\mathbb {C}}_{\langle n\rangle , N}$ instead of ${\mathbb {C}}_{\langle n\rangle }$.

So far, $\mathcal {A}_n$ is only a vector space and $\boxtimes$ is just a symbol which will be different from $\otimes$ when we leave the category of vector spaces and grasp $\mathcal {A}_n$ already as an algebra. The bilinearity of $\boxtimes$ is due to the coupling constants ${\bar{g}}$ of interaction vertices $O={{\,\mathrm{Tr}\,}}_{N}[ {\bar{g}} Q_1 ]{{\,\mathrm{Tr}\,}}_{N}Q_2 = {{\,\mathrm{Tr}\,}}_{N}Q_1 {{\,\mathrm{Tr}\,}}_{N}[ {\bar{g}} Q_2 ]$, which can “enter into any trace”. Thus, $\boxtimes$ must satisfy $(\lambda U )\boxtimes W = U \boxtimes ( \lambda W)$ for complex $\lambda$ and $U,W\in {\mathbb {C}}_{\langle n\rangle }$. The noncommutative Hessian can be extended to products of traces as follows:

#### Definition 4

On double traces ${{\,\mathrm{Hess}\,}}: {\text {im}}{{\,\mathrm{Tr}\,}}^{\otimes 2} \rightarrow M_n(\mathcal {A}_n )$ is given by

33 $$\begin{aligned} {{\,\mathrm{Hess}\,}}\big \{\!{{\,\mathrm{Tr}\,}}^{\otimes 2} (P\otimes Q)\big \} ={{\,\mathrm{Hess}\,}}P \times {{\,\mathrm{Tr}\,}}Q + {{\,\mathrm{Hess}\,}}Q \times {{\,\mathrm{Tr}\,}}P + \Delta (P,Q)\,, \end{aligned}$$

where $\Delta (P,Q)=(\Delta _{a,b}(P,Q))_{a,b=1,\ldots ,n}$ has the following $M_n$-matrix entries:

34 $$\begin{aligned} \Delta _{a,b}(P,Q)&=\partial _{X_a}{{\,\mathrm{Tr}\,}}_{N}P \boxtimes \partial _{X_b}{{\,\mathrm{Tr}\,}}_{N}Q + \partial _{X_a}{{\,\mathrm{Tr}\,}}_{N}Q \boxtimes \partial _{X_b} {{\,\mathrm{Tr}\,}}_{N}P \nonumber \\&={{\,\mathrm{{\mathscr {D}}}\,}}_{X_a} P \boxtimes {{\,\mathrm{{\mathscr {D}}}\,}}_{X_b} Q + {{\,\mathrm{{\mathscr {D}}}\,}}_{X_a} Q \boxtimes {{\,\mathrm{{\mathscr {D}}}\,}}_{X_b} P\,. \end{aligned}$$

#### Lemma 1

The trace ${{\,\mathrm{Tr}\,}}_{\mathcal {A}_n}$ on $\mathcal {A}_n$ is defined^12^ in terms of ${{\,\mathrm{Tr}\,}}_{N}$ by linear extension of

35a $$\begin{aligned} {{\,\mathrm{Tr}\,}}_{\mathcal {A}_n} (P\otimes Q)&= {{\,\mathrm{Tr}\,}}_N^{\otimes 2} (P\otimes Q) = {{\,\mathrm{Tr}\,}}_{N}(P) \times {{\,\mathrm{Tr}\,}}_{N}(Q) \end{aligned}$$

35b $$\begin{aligned} {{\,\mathrm{Tr}\,}}_{\mathcal {A}_n} (P\boxtimes Q)&= {{\,\mathrm{Tr}\,}}_{N}(PQ) \end{aligned}$$

#### Proof

The tadpoles yield the desired relations. To obtain the first, for any fixed $c\in \{1,\ldots ,n\}$, consider an interaction vertex $O={\bar{g}} {{\,\mathrm{Tr}\,}}(X_c P X_c Q)$ with $P,Q\in {\mathbb {C}}_{\langle n\rangle }$ satisfying $\partial _{X_c}P=\partial _{X_c} Q=0$ (e.g., take $P,Q \in {\mathbb {C}}_{\langle n-1 \rangle }= {\mathbb {C}}\langle X_1,\ldots , X_{c-1}, X_{c+1},\ldots , X_n\rangle$). The contribution to the rhs of the flow equation is

36 $$\begin{aligned} \frac{1}{2} \mathrm {STr}{{\,\mathrm{Hess}\,}}O = \frac{{\bar{g}}}{2} {{\,\mathrm{Tr}\,}}_{\mathcal {A}_n}(P\otimes Q+ Q\otimes P) + \text {terms without }c\text {-propagators}\,. \end{aligned}$$

The value of the first two summands is determined by the effective vertex of the graphs that the Hessian computes according to Eq. (8). These are such that the two ribbons are attached at the only two $X_c$ matrices in *O*,The ellipsis means that in the graphs, *P* is the word after the contracted $X_c$ running clockwise until the next $X_c$, after which *Q* begins. The seemingly different propagator contraction is just an attempt to reflect that in the first graph *P* is inside the loop and *Q* outside, with these words in the other way around for the second graph. However, these two graphs are indistinguishable, thus, for each graph the effective vertex reads ${\bar{g}} {{\,\mathrm{Tr}\,}}_{N}(P) \times {{\,\mathrm{Tr}\,}}_{N}(Q)$, so by Eqs. (36), (35a) follows. To obtain the other product, we consider tadpoles with the ends of the propagator on different traces of the same operator. Letwhose Hessian (*cc*)-entry readsBy Eqs. (33) and (34),

37 $$\begin{aligned} \frac{1}{2} \mathrm {STr}{{\,\mathrm{Hess}\,}}O = \frac{{\bar{g}}'}{2} {{\,\mathrm{Tr}\,}}_{\mathcal {A}_n}(P\boxtimes Q+ Q\boxtimes P) + \text {terms without } c\text {-propagators}\,. \end{aligned}$$

Ignoring the two coupling, the effective vertex of each graph is ${{\,\mathrm{Tr}\,}}_{N}(PQ)$, which must be the value of ${{\,\mathrm{Tr}\,}}_{\mathcal {A}_n}(P\boxtimes Q)$, but since the graphs are indistinguishable, also of ${{\,\mathrm{Tr}\,}}_{\mathcal {A}_n}(Q\boxtimes P)$, Therefore, Eq. (37) implies Eq. (35b).

Now let us consider the general case, where *P* might depend on $X_c$ (the dependence of *Q* on $X_c$ can be likewise implemented, additionally, but the argument is the same in essence). Suppose that $P=P_{\text {L}} X_c P_{\text {R}}$, where $P_{\text {L}},P_{\text {R}} \in {\mathbb {C}}_{\langle n\rangle }$ are monomials independent of $X_c$. In this simple case, the rhs of Eq. (36) receives the correction ${\bar{g}} {{\,\mathrm{Tr}\,}}_{\mathcal {A}_n}[ P_{\text {L}} \otimes P_{\text {R}}X_c Q+ P_{\text {R}}X_c Q\otimes P_{\text {L}} ]$, by the formula (8) for the Hessian. However, since *P*, *Q* are arbitrary, these terms cannot contribute to the coefficient of ${{\,\mathrm{Tr}\,}}_{N}P {{\,\mathrm{Tr}\,}}_{N}Q$ in $\frac{1}{2} \mathrm {STr}({{\,\mathrm{Hess}\,}}O)$, since none of the graphs in such correction comply with having effective vertex (proportional to) ${{\,\mathrm{Tr}\,}}_{N}P {{\,\mathrm{Tr}\,}}_{N}Q$, and the word *P* has been split. Therefore, such contributions can be ignored (they do contribute, but to other effective vertices). A similar treatment for a generic word *P* and *Q* that might contain $X_c$ concludes also the proof of (37) without restrictions on *P* and *Q* imposed above. $\square$

In order to justify Eq. (29), we now define both ${\mathcal {C}}$ and $\Gamma {}^{\textsc {Int}}_N [{\mathbb {X}}]$ by

38 $$\begin{aligned} R_N+{{\,\mathrm{Hess}\,}}\Gamma _N [{\mathbb {X}}] =:{\mathcal {C}}{}^{-1}+ {{\,\mathrm{Hess}\,}}\Gamma {}^{\textsc {Int}}_N [{\mathbb {X}}]\,, \end{aligned}$$

where $\Gamma {}^{\textsc {Int}}_N [{\mathbb {X}}]$ contains only interaction vertices (and $[{{\,\mathrm{Tr}\,}}_{N}X_c]^2$ counts as such; ${\mathcal {C}}$ is the correlation or inverse propagator). That is, $\Gamma {}^{\textsc {Int}}_N$ is defined in such a way that the Gaussian part $\mathrm {d}\gamma {}^{\mathrm {eff}}_N$ in the effective measure is factorized out:

39a $$\begin{aligned} \mathrm {d}\mu {}^{\mathrm {eff}}_N( {\mathbb {X}} )&= \mathrm {e}^{- \Gamma _N [{\mathbb {X}}] } \mathrm {d}\mathbb {X}{}_{\textsc {Leb}}= \mathrm {e}^{- \Gamma {}^{\textsc {Int}}_N [{\mathbb {X}}] } \mathrm {d}\gamma {}^{\mathrm {eff}}_N({\mathbb {X}})\,, \end{aligned}$$

39b $$\begin{aligned} \mathrm {d}\gamma {}^{\mathrm {eff}}_N({\mathbb {X}})&= \prod _{c=1}^n \mathrm {e}^{- Z_c {{\,\mathrm{Tr}\,}}_{N}(X^2_c/2)} (\mathrm {d}X_c){}_{\textsc {Leb}}\,. \end{aligned}$$

Notice that one could have been tempted, inspired by [28], to separate the Hessian in its field-independent part (defined by its vanishing when ${\mathbb {X}}=0$) and the field dependent part as performed in the functional renormalization treatment to one-matrix models by [26]. The “field part” of the algebra $\mathcal {A}_{n,N}$ consists of non-trivial words (i.e., except multiples of $1_N\otimes 1_N$ and $1_N\boxtimes 1_N$). But the presence of double-trace quadratic operators $\frac{1}{2}[{{\,\mathrm{Tr}\,}}(X_c)]^2$, whose Hessian is $\frac{1}{2}{{\,\mathrm{Hess}\,}}\{ ({{\,\mathrm{Tr}\,}}X_c)^2\} = \mathrm {diag}_n [0,\ldots , 1_N\boxtimes 1_N ,0,\ldots , 0]$, with the non-zero in the (*cc*)th entry of the $M_n$-block diagonal $\mathrm {diag}_n$, lies in the field-independent part, and this impairs (as we see now) the Neumann expansion. On the other hand, the definition (38) guarantees that the propagator ${\mathcal {C}}{}^{-1}$ is $1_n \otimes 1_N\otimes 1_N$ times a function (on $[1,\ldots , N]^2$), due to

$$\begin{aligned} \sum _{c=1}^n {{\,\mathrm{Hess}\,}}\Big \{\frac{1}{2} {{\,\mathrm{Tr}\,}}(X_c^2)\Big \}= \sum _{c=1}^n \mathrm {diag}_n [0,\ldots , \underbrace{1_N \otimes 1_N }_{c\text {th place}},0,\ldots , 0] = 1_n\otimes 1_N\otimes 1_N\,. \end{aligned}$$

When the wave function renormalization constant $Z_c$ is supposed to be equal for all matrices, $Z_c=Z$, then ${\bar{h}}_k(N,\eta )$, $\eta =-\partial _t \log Z$, and the sums in ${\bar{h}}_k$ can be approximated by integrals of the form $\frac{1}{N^2}\int (\partial _t r_N)_{\sigma ,\tau } {\mathcal {C}}^{k+1}_{\tau ,\sigma }\mathrm {d}\sigma \, \mathrm {d}\tau$ that remain finite as $N\rightarrow \infty$. We do not study the space of possible regulators (in itself, interesting), but we stress that the expansion (29) in unitary invariants is an assumption. Ideally, as commented in [26], since $R_N$ breaks the symmetry, Eq. (29) should include operators $\mathrm {STr}( \partial _t R_N {\mathcal {C}} [ {{\,\mathrm{Hess}\,}}\Gamma _N{}^{\textsc {Int}}[{\mathbb {X}}] {\mathcal {C}} ]^{\star k})$. However, identifying these operators with broken unitary symmetry is out of our present scope and for now the best one can do is to split, as in Eq. (29), the rhs of Wetterich equation in $R_N$-dependent and $R_N$-independent part. The main result of this article is the unique description of the latter.

#### Theorem 2

For multiple-trace self-adjoint *n*-matrix ensembles, assume the rhs of Wetterich equation to be computable in terms of $\mathrm U(N)$-invariants as the geometric series (29) in the Hessian. Moreover, require that in Eq. (29) only one-loop graphs are generated. Then, the powers $({{\,\mathrm{Hess}\,}}\Gamma {}^{\textsc {Int}}_N [{\mathbb {X}}])^{\star k}$ are taken in the algebra $M_n({\mathcal {A}}_{n,N},\star )$ of $n\times n$ matrices with entries in ${\mathcal {A}}_{n,N}$, explicitly

40 $$\begin{aligned} M_n({\mathcal {A}}_{n,N}) = M_n({\mathbb {C}})\otimes {\mathcal {A}}_{n,N}\,,\quad {\mathcal {A}}_{n,N}= {\mathbb {C}}_{\langle n\rangle , N}^{\,\otimes \, 2} \oplus {\mathbb {C}}_{\langle n\rangle , N}^{\,\boxtimes \, 2}\,, \end{aligned}$$

whose product is given entry-wise by $({\mathcal {P}} \star {\mathcal {Q}})_{a,c} = \sum _{b=1}^n P_{a,b} \star Q_{b,c}$ for ${\mathcal {P}}=(P_{a,b})_{a,b=1,\ldots , n},$ and $\mathcal Q=(Q_{a,b})_{a,b=1,\ldots , n} \in M_n({\mathcal {A}}_{n,N})$, and each entry $P_{a,b}$ and $Q_{b,c}$ obeys the following multiplication rule, given here on homogeneous elements of ${\mathcal {A}}_{n,N}$: for any $P,Q,U,W \in {\mathbb {C}}_{\langle n\rangle }$,

41a $$\begin{aligned} (U \otimes W) \ \star \ ( P\otimes Q)&= PU \otimes WQ \,, \end{aligned}$$

41b $$\begin{aligned} (U\boxtimes W) \ \star \ ( P\otimes Q)&=U \boxtimes PWQ \,, \end{aligned}$$

41c $$\begin{aligned} (U \otimes W) \ \star \ ( P\boxtimes Q)&= WPU \boxtimes Q \,, \end{aligned}$$

41d $$\begin{aligned} (U\boxtimes W) \ \star \ ( P\boxtimes Q)&= {{\,\mathrm{Tr}\,}}_{N}(WP) U\boxtimes Q \,. \end{aligned}$$

#### Proof

Sect. 3 is the proof. $\square$

In other words, if one computes functional renormalization of matrix models with a product different from Eq. (41), *either* contributions that do not have the one-loop structure appear in the $\beta$-functions (14), *or* it is impossible to compute the renormalization flow by splitting, in regulator-dependent and regulator-independent parts as in (29)—regardless of what ${\bar{h}}_k$ might be.

#### Remark 1

There are two interesting limiting cases,^13^ large-*N* (together with the initial scale of the bare action $N_\infty \rightarrow \infty$) and small-*N*. From Fig. 1, it is evident that *N*-factors appear only when one-loop graphs have the “empty word” $1_N$ at any side. This suggests that the algebra of Theorem 2 could be reduced to Eq. (41a), but actually double-traces appear again in Eq. (41d), and ${{\,\mathrm{Tr}\,}}_{N}(Q_1)\times {{\,\mathrm{Tr}\,}}_{N}(Q_2)$ compete with terms of the form $N{{\,\mathrm{Tr}\,}}_{N}(P)$. Further, this argument should be thoroughly investigated, since the ensemble in the large-*N* depends also on the power-counting, that is, on the solution for the $\kappa _\alpha$ and $\lambda _\alpha$; see the discussion just above Eq. (14). For $\beta$-functions computed with the algebra Eq. (41), see [3, Thm. 7.2] and [24]. The critical behavior could be explored in the sense of [26] as eigenvalues of the stability matrix, namely , where the bullet means the fixed-point solutions of the system (14),  and  for all interactions $\alpha$ and all matrices $c=1,\ldots ,n$. In the large-*N*, for the two-matrix model with 48 operators (that is the number of operators in a sextic truncation) compatible with the symmetries of the *ABAB*-model, the unique fixed point solution with a single positive eigenvalue of the stability matrix happens when two coupling constants have the value 0.07972 ($1/4\pi = 0.07957\ldots$ is the critical value for the coupling constants in [5], when one takes their sign and normalization conventions) and some double-trace operators like ${{\,\mathrm{Tr}\,}}^2_N(A),{{\,\mathrm{Tr}\,}}_{N}^2(A^2)$, ${{\,\mathrm{Tr}\,}}_{N}(A)\times {{\,\mathrm{Tr}\,}}_{N}(B^3)$, do contribute to the flow (at least so with the regulator of App. A). The limit $N\rightarrow 1$ ($t\rightarrow 0$) should yield the full effective action (see limits in App. A), but this is unexplored here and needs an independent study. In the worst of the cases, the full algebra (41) is needed to next-to-leading-order or nlo corrections, but bounds on those nlo-terms are precisely the beginning of an analytic approach.

#### Remark 2

(The product $\star$ in terms of matrix entries) Consider the permutation $\tau =(13)\in \mathrm {Sym}(4)$ and denote by $\mathrm {id}$ the identity of the symmetric group $\mathrm {Sym}(4)$. Let $\rho ,\pi \in \{ \mathrm {id}, \tau \}$. Then, if $a,b,c,d=1,\ldots ,N$, and $Y_1,Y_2,Y_3,Y_4\in {\mathbb {C}}_{\langle n\rangle }\subset M_N({\mathbb {C}})$ are monomials, the four products of Theorem 2 are summarized in the following equation, where the sum over $x,y=1,\ldots ,N$ is implicit:

42 $$\begin{aligned} (Y_1 \otimes _\rho Y_2) \star (Y_3\otimes _\pi Y_4)_{ab;cd}= (Y_1)_{\rho (a)\rho (b)} (Y_2)_{\rho (x)\rho (y)} (Y_3)_{\pi (y)\pi (x)} (Y_4)_{\pi (c)\pi (d)} \end{aligned}$$

where for $\varpi \in \{ \mathrm {id}, \tau \}$, $\otimes _\varpi =\otimes$ if $\varpi =\tau =(13)$ and $\otimes _\varpi =\otimes _{\mathrm {id}}=\boxtimes$ if $\varpi$ is the trivial permutation.. Also $\rho$ acts as element of $\mathrm {Sym}(a,b,x,y)$ and $\pi$ on $\mathrm {Sym}(y,x,c,d)$. For instance, in the nontrivial case $\rho =\tau$, $\tau (a,b,x,y)=(x,b,a,y)$. We remark that in order to keep the Hessian simple in this paper, the convention is the opposite of [3], i.e., $\otimes _\tau$ there is $\otimes$ here; and the $\otimes$ of [3] corresponds to the $\boxtimes$ here. The particular permutation $\tau =(13)$ might seem at first arbitrary, but it is actually natural and can be found in *op.cit.* or in [6, Eq. 5].

## The proof of the main statement

**Fig. 4:**
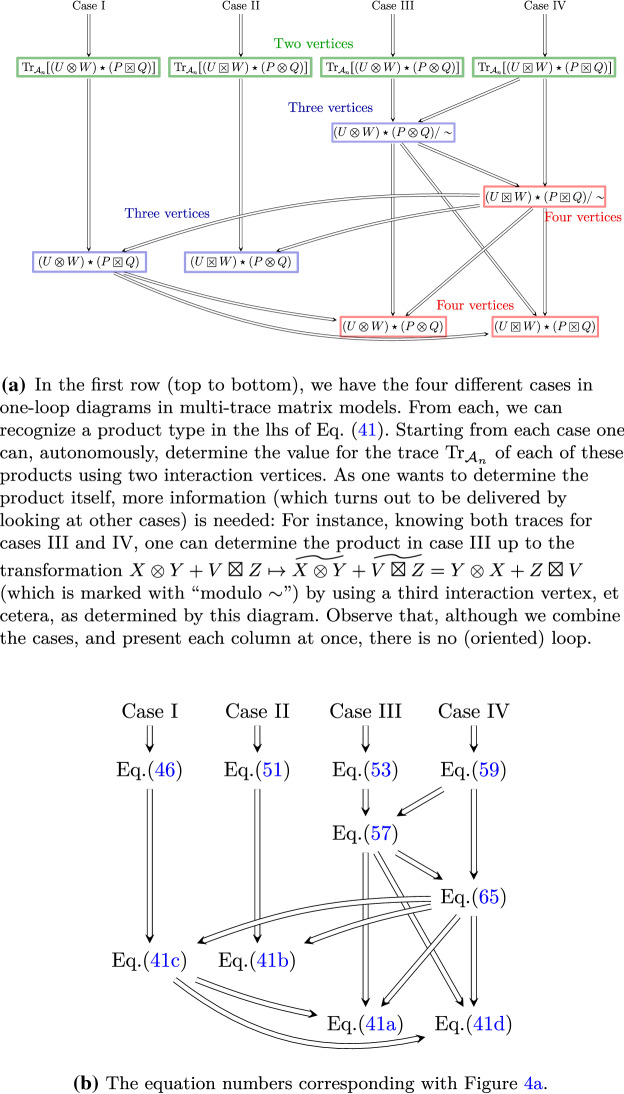
The “topology” of the proof of Theorem 2 showing the absence of logic loops, notwithstanding the mix of cases in the proof. The arrows are implications. These diagrams show how we “bootstrap” the algebra

Figure 4 gives the logic structure in the proof. By “$s \subset {{\,\mathrm{Hess}\,}}_{a,b} ( O)$” we abbreviate that *s* is a summand in ${{\,\mathrm{Hess}\,}}_{a,b} (O)$. Further, $M,L,P,Q,R,S,T,U,V,W\in {\mathbb {C}}_{\langle n\rangle }$ are arbitrary monomials.

### Proof of Theorem 2

Start with the *k*th power of a Hessian. First, we argue that we can simplify this situation and deduce the behavior regarding the *k*th power for any *k* from the square of a Hessian. Supertraces of products of Hessians will be sums over terms of the following form:43The associativity of the product $\star$ follows from the definition of effective vertices (but should be verified purely algebraically, after the product is constructed):44where the gray boxes with uncontracted, protruding ribbon edges mean the new interactions formed from the two grouped interaction vertices. The new cyclic order is determined by the propagator, together with the half-edges it is attached to, being shrunk. The left corresponds to the $[{{\,\mathrm{Hess}\,}}_{a,b} (O_1) \star {{\,\mathrm{Hess}\,}}_{b,c} (O_2) ] \star {{\,\mathrm{Hess}\,}}_{c,d}(O_3)$ bracketing, while the right one to ${{\,\mathrm{Hess}\,}}_{a,b} (O_1) \star [ {{\,\mathrm{Hess}\,}}_{b,c} (O_2) \star {{\,\mathrm{Hess}\,}}_{c,d}(O_3)]$.

We have four cases, depending on the way the four propagators in the loop connect the interaction vertices of $k=2$ interaction vertices. The fact that ${\mathscr {A}}=M_n(\mathcal {A}_n)$ is an associative algebra (or recursive application of (44)) allows us not to consider more cases. However, to determine the product, $k=3,4$ will yield also useful information too.

- Case I: *When two ribbons in the loop lie in the same trace in the first interaction vertex, but in different traces in the second:*45 Suppose that the interaction vertices have one and two traces, respectively. In fact they might have more traces, but these not being implied in the loop for the present case, they remain intact; thus, we do not loose generality by this simplification. There exist then words *T*, *U*, *V*, *W* (which might be empty) such that
  $$\begin{aligned} O_1 = {\bar{g}}_1 {{\,\mathrm{Tr}\,}}(UX_a W X_b )\quad \text{ and }\quad O_2={\bar{g}}_2 {{\,\mathrm{Tr}\,}}( X_b T) {{\,\mathrm{Tr}\,}}( X_a V) \,. \end{aligned}$$
  The words $T,U,V,W\in {\mathbb {C}}_{\langle n\rangle }$ might contain the letters $X_a, X_b$, but we are analyzing only the summand in the lhs of (45). To compute the contribution of the two Hessians to this precise summand, we get by Eq. (8)
  $$\begin{aligned} {{\,\mathrm{Hess}\,}}_{a,b} O_1 \supset {\bar{g}}_1 \partial _{X_a} \circ \partial _{X_b } {{\,\mathrm{Tr}\,}}(U X_a W X_b) = {\bar{g}}_1 U\otimes W\,, \end{aligned}$$
  and by Eq. (34),
  $$\begin{aligned} {{\,\mathrm{Hess}\,}}_{b,a} O_2 \supset {\bar{g}}_2 {{\,\mathrm{{\mathscr {D}}}\,}}_{X_b} {{\,\mathrm{Tr}\,}}( X_b T) \boxtimes {{\,\mathrm{{\mathscr {D}}}\,}}_{X_a} {{\,\mathrm{Tr}\,}}( X_a V) = {\bar{g}}_2 T\boxtimes V \,. \end{aligned}$$
  Now, since the effective vertex of (45) is formed by shrinking the green and red propagators and merging the rest of the ribbon half-edges while preserving the order, the graph (45) implies that the effective vertex is ${\bar{g}}_1 {\bar{g}}_2{{\,\mathrm{Tr}\,}}_{N}(W T U V )$. By Wetterich equation,
  46 $$\begin{aligned} {{\,\mathrm{Tr}\,}}_{\mathcal {A}_n}[ (U\otimes W) \star (V\boxtimes T) ] = {{\,\mathrm{Tr}\,}}_{N}(W T U V ) \,. \end{aligned}$$
  Since the lhs is a single trace, this is enough to conclude that the result of $(U\otimes W) \star (V\boxtimes T)$ must be “a $\boxtimes$ inserted somewhere in the cyclic word *WTUV*”, otherwise it would be a product of the form $w_1\otimes w_2$ which, when traced, would yield a *N*-factor, in case that any of the words $w_1$ or $w_2$ is trivial, and a double trace if both are not trivial. We also know that the result of $(U\otimes W) \star (V\boxtimes T)$ must be an ordinary and not a cyclic word; thus, so far, we need to know how to root it, i.e., the expression for $(U\otimes W) \star (V\boxtimes T)$ should be listed in
  47 $$\begin{aligned} 1&\boxtimes WTUV ,&W&\boxtimes TUV,&WT&\boxtimes UV,&WTU&\boxtimes V,&WTUV&\boxtimes 1\,, \nonumber \\ 1&\boxtimes TUVW,&T&\boxtimes UVW,&TU&\boxtimes VW,&TUV&\boxtimes W,&TUVW&\boxtimes 1\,, \nonumber \\ 1&\boxtimes UVWT,&U&\boxtimes VWT,&UV&\boxtimes WT,&UVW&\boxtimes T,&UVWT&\boxtimes 1\,, \nonumber \\ 1&\boxtimes VWTU,&V&\boxtimes WTU,&VW&\boxtimes TU,&VWT&\boxtimes U,&VWTU&\boxtimes 1\,.\qquad \qquad \qquad \end{aligned}$$
  To discard the wrong ones, we first consider the following interaction vertices:
  $$\begin{aligned} O_1&= {\bar{g}}_1 {{\,\mathrm{Tr}\,}}_{N}(X_bW X_a U)\,, \\ O_2&= {\bar{g}}_2 {{\,\mathrm{Tr}\,}}_{N}(X_b T) {{\,\mathrm{Tr}\,}}_{N}( X_c V) \,,\\ O_3&={\bar{g}}_3 {{\,\mathrm{Tr}\,}}_{N}(X_c R) {{\,\mathrm{Tr}\,}}_{N}(S X_a)\,. \end{aligned}$$
  and the corresponding product of Hessians of each of these (in that order), which contains in particular, the next graph:  The effective vertex must be ${{\,\mathrm{Tr}\,}}_{N}(VWSU) {{\,\mathrm{Tr}\,}}_{N}(RT)$, so
  48 $$\begin{aligned} {{\,\mathrm{Tr}\,}}_{\mathcal {A}_n}\big \{ [( W\otimes U ) \star (V\boxtimes T)] \star (R\boxtimes S)\big \} = {{\,\mathrm{Tr}\,}}_{N}(VWSU) \times {{\,\mathrm{Tr}\,}}_{N}(RT)\,. \end{aligned}$$
  One can use the previous graph to discard elements in the list (47). For instance, we suppose that $( W\otimes U ) \star (V\boxtimes T) = W \boxtimes TUV$. For the product inside curly brackets $\{ \ldots \}$, using Eq. (60c) or Eq. (60d) (equivalently, Eq. (65); Fig. 4b), one gets the following possibilities:
  49 $$\begin{aligned} ={\left\{ \begin{array}{ll} S\boxtimes W {{\,\mathrm{Tr}\,}}_{N}(RTUV) &{} \text {if Eq. } (60c) \text {holds}\,, \\ W\boxtimes S {{\,\mathrm{Tr}\,}}_{N}(RTUV) &{} \text {if Eq. } (.60d) \text {holds}\,. \end{array}\right. } \end{aligned}$$
  But the trace of it yields in either case ${{\,\mathrm{Tr}\,}}_{N}(SW) {{\,\mathrm{Tr}\,}}_{N}(RTUV)$ which differs from Eq. (48). Thus, $( W\otimes U ) \star (V\boxtimes T) = W \boxtimes TUV$ is impossible. By the same token, with the same counterexample above, one discards the possibilities that do not contain a factor of the empty word 1, except $( W\otimes U ) \star (V\boxtimes T) =UVW\boxtimes T$.
  Regarding those possibilities containing the factor of 1, following any of the prescription of the leftmost column in (47) for the square brackets product, and the Case IV, which is to say either Eq. (60c) or Eq. (60d), for the resulting multiplication of the form $w_1\boxtimes w_2 \star w_3\boxtimes w_4$, one easily sees that these generate a factor ${{\,\mathrm{Tr}\,}}_{N}(S)$; likewise, those possible products on the rightmost columns (47) generate a factor ${{\,\mathrm{Tr}\,}}_{N}(R)$. Both lead then to contradiction with the previous graph. Therefore, indeed $( W\otimes U ) \star (V\boxtimes T) =UVW\boxtimes T$, i.e., Eq. (41c) holds.
- Case II: *When two ribbons in the loop lie in the same trace in the first interaction vertex, but in different traces in the second:* This case is proven by swapping the roles of the first and second interaction vertices in Case I. Since the proof is analogous, we rather sketch it. The next graph can be used 50 for suitable operators $O_1$ and $O_1$ (constructed in a similar way to Case I) to obtain
  51 $$\begin{aligned} {{\,\mathrm{Tr}\,}}_{\mathcal {A}_n}\big ( U\boxtimes W \star P\otimes Q \big ) = {{\,\mathrm{Tr}\,}}_{N}(QUPW)\,. \end{aligned}$$
  From Eqs. (60c) and (60d) (see Fig. 4) and the graph  one can create suitable operators $O_1,O_2,O_3$ to deduce Eq. (41b).
- Case III:* When two ribbons in the loop lie on the same trace in both the first and second interaction vertices:*52 We consider operators $O_1= {\bar{g}}_1 {{\,\mathrm{Tr}\,}}_{N}(X_aW X_b U)$ and $O_2= {\bar{g}}_2 {{\,\mathrm{Tr}\,}}_{N}(X_a P X_b Q)$. These might have more traces, but as depicted above, these being outside the loop, do not suffer any transformation (in that summand) and can be ignored. Then, ${{\,\mathrm{Hess}\,}}_{a,b} O_1 \star {{\,\mathrm{Hess}\,}}_{b,a} O_2 = (U\otimes W) \star (P\otimes Q)$. According to Wetterich equation, the effective vertex must be
  53 $$\begin{aligned} {{\,\mathrm{Tr}\,}}_{\mathcal {A}_n}[(U\otimes W) \star (P\otimes Q)] ={{\,\mathrm{Tr}\,}}_{N}(PU) {{\,\mathrm{Tr}\,}}_{N}(WQ). \end{aligned}$$
  which implies either of the following possibilities:
  54a $$\begin{aligned} (U\otimes W)\ \star \ (P\otimes Q)&= {{\,\mathrm{Tr}\,}}_{N}(PU) W\boxtimes Q \end{aligned}$$
  54b $$\begin{aligned} (U\otimes W)\ \star \ (P\otimes Q)&= {{\,\mathrm{Tr}\,}}_{N}(PU) Q\boxtimes W \end{aligned}$$
  54c $$\begin{aligned} (U\otimes W) \ \star \ (P\otimes Q)&= PU \otimes QW \end{aligned}$$
  54d $$\begin{aligned} (U\otimes W) \ \star \ (P\otimes Q)&= UP \otimes QW \end{aligned}$$
  54e $$\begin{aligned} (U\otimes W) \ \star \ (P\otimes Q)&= UP \otimes WQ \end{aligned}$$
  54f $$\begin{aligned} (U\otimes W) \ \star \ (P\otimes Q)&= PU \otimes WQ \end{aligned}$$
  To obtain the right one(s), we consider now the third power of the Hessian, but in the contraction with the additional vertex Case IV shall be here useful. By contradiction to each of the cases, we suppose that Eq. (54a) holds. Then, consider the following interaction vertices:
  $$\begin{aligned} O_1&= {\bar{g}}_1 {{\,\mathrm{Tr}\,}}_{N}(X_aW X_b U)\,, \\ O_2&= {\bar{g}}_2 {{\,\mathrm{Tr}\,}}_{N}(X_b Q X_c P) \,,\\ O_3&={\bar{g}}_3 {{\,\mathrm{Tr}\,}}_{N}(X_c R) {{\,\mathrm{Tr}\,}}_{N}(S X_a)\,. \end{aligned}$$
  By a similar ribbon graph argument, we obtain, using the hypothesis, that $({{\,\mathrm{Hess}\,}}_{a,b} O_1 \star {{\,\mathrm{Hess}\,}}_{b,c} O_2 )\star {{\,\mathrm{Hess}\,}}_{c,a} O_3$ which is, modulo the coupling constants $[(U\otimes W) \star (P\otimes Q)]\star (R\boxtimes S)= [{{\,\mathrm{Tr}\,}}_{N}(PU) W\boxtimes Q] \star (R\boxtimes S)$. Applying ${{\,\mathrm{Tr}\,}}_{\mathcal {A}_n}$ to this quantity we deduce, according to the partial conclusion in Case IV (recalling that the bracketing is irrelevant due to Eq. (44)), that
  55 $$\begin{aligned} {{\,\mathrm{Tr}\,}}_{\mathcal {A}_n}\big \{ (U\otimes W) \star (P\otimes Q) \star (R\boxtimes S)\big \}&= {{\,\mathrm{Tr}\,}}_{N}(PU) {{\,\mathrm{Tr}\,}}_{\mathcal {A}_n}\big \{ (W\boxtimes Q ) \star (R\boxtimes S)\big \} \nonumber \\&={{\,\mathrm{Tr}\,}}_{N}(PU) {{\,\mathrm{Tr}\,}}_{N}(QR) {{\,\mathrm{Tr}\,}}_{N}(WS). \end{aligned}$$
  However, by looking at the graph that the product in curly brackets represents, the previous equation cannot be true, for the graph leads to a single trace, namely ${{\,\mathrm{Tr}\,}}_{N}(WQRPUS)$. This is a contradiction with the supposition that $(U\otimes W) \star (P\otimes Q) = {{\,\mathrm{Tr}\,}}_{N}(PU) W\boxtimes Q$. Hence, we discard Eq. (54a). By the same argument in number of traces, we discard also Eq. (54b). Further, with the next counterexample 56 obtained from the Hessians of the operators
  $$\begin{aligned} O_1= {\bar{g}}_1 {{\,\mathrm{Tr}\,}}_{N}(X_aW X_b U)\,, \,\, O_2 = {\bar{g}}_2 {{\,\mathrm{Tr}\,}}_{N}(X_b Q X_c P)\,,\,\, O_3= {\bar{g}}_3 {{\,\mathrm{Tr}\,}}_{N}(X_a R X_c S )\, . \end{aligned}$$
  Then, two further possibilities are ruled out, for, on the one hand, Eq. (54c) implies that the effective vertex is ${{\,\mathrm{Tr}\,}}_{N}(RPU){{\,\mathrm{Tr}\,}}_{N}(SQW)$; and Eq. (54e), on the other hand, implies that it is ${{\,\mathrm{Tr}\,}}_{N}(UPR){{\,\mathrm{Tr}\,}}_{N}(WQS)$, modulo coupling constants. Either is different from the effective vertex for the graph (56), namely ${{\,\mathrm{Tr}\,}}_{N}( WQS ){{\,\mathrm{Tr}\,}}_{N}(RPU)$. So only the next two are possible:
  57 $$\begin{aligned} (U\otimes W) \star (P\otimes Q) = UP \otimes QW \qquad (U\otimes W) \star (P\otimes Q) = PU \otimes WQ\,. \end{aligned}$$
  We solve now Case IV and then determine which of the two is the right expression.
- Case IV: *When two ribbons in the loop lie on different traces in both interaction vertices:*58 Notice that, notwithstanding the disconnectedness of the ribbons in this graph $G_{\textsc {iv}}$, as a graph in the field theory context, what matters is the connectivity of its skeleton $G_{\textsc {iv}}^\circ$ (${\bar{g}}_i$ are the coupling constants for both traces inside the dashed circle, cf. Def 2). We construct now operators that yield the desired product. Let $O_1= {\bar{g}}_1 {{\,\mathrm{Tr}\,}}_{N}(UX_a) {{\,\mathrm{Tr}\,}}_{N}(X_b W)$ and $O_2= {\bar{g}}_2 {{\,\mathrm{Tr}\,}}_{N}(QX_a) {{\,\mathrm{Tr}\,}}_{N}(X_b P)$. Then, the product of Hessians in Eq. (58) contains $(U\boxtimes W) \star ( P\boxtimes Q)$ as a summand. The effective vertex must be what we obtain by shrinking the propagators. In turn, in the rhs of Wetterich Eq. (28) this effective vertex is obtained by tracing over^14^$\mathcal {A}_n$, so
  59 $$\begin{aligned} {{\,\mathrm{Tr}\,}}_{\mathcal {A}_n}[(U\boxtimes W) \star ( P\boxtimes Q)] = {{\,\mathrm{Tr}\,}}_{N}( WP ) \times {{\,\mathrm{Tr}\,}}_{N}(UQ ) \,. \end{aligned}$$
  This means that the quantity in square brackets must be either of the following product formulas for $\star$:
  60a $$\begin{aligned} (U\boxtimes W) \ \star \ ( P\boxtimes Q)&= P\boxtimes W {{\,\mathrm{Tr}\,}}_{N}(QU) \end{aligned}$$
  60b $$\begin{aligned} (U\boxtimes W) \ \star \ ( P\boxtimes Q)&= W\boxtimes P {{\,\mathrm{Tr}\,}}_{N}(QU) \end{aligned}$$
  60c $$\begin{aligned} (U\boxtimes W)\ \star \ ( P\boxtimes Q)&= Q\boxtimes U {{\,\mathrm{Tr}\,}}_{N}(PW) \end{aligned}$$
  60d $$\begin{aligned} (U\boxtimes W) \ \star \ ( P\boxtimes Q)&= U\boxtimes Q {{\,\mathrm{Tr}\,}}_{N}(PW) \end{aligned}$$
  60e $$\begin{aligned} (U\boxtimes W) \ \star \ ( P\boxtimes Q)&= PW \otimes QU \end{aligned}$$
  60f $$\begin{aligned} (U\boxtimes W) \ \star \ ( P\boxtimes Q)&= WP \otimes QU \end{aligned}$$
  60g $$\begin{aligned} (U\boxtimes W) \ \star \ ( P\boxtimes Q)&= WP \otimes UQ \end{aligned}$$
  60h $$\begin{aligned} (U\boxtimes W) \ \star \ ( P\boxtimes Q)&= PW \otimes UQ \end{aligned}$$
  To determine the correct product, we consider higher powers of the Hessian, and one of the partial conclusion of the Case III, Eq. (53).
  By contradiction, suppose that Eq. (60a) holds. Then applying twice this equation,
  61 $$\begin{aligned}{}[(U\boxtimes W) \star ( P\boxtimes Q) ] \star (R\boxtimes S)&= {{\,\mathrm{Tr}\,}}_{N}(QU) P \boxtimes W \star R\boxtimes S \nonumber \\&= {{\,\mathrm{Tr}\,}}_{N}(QU) {{\,\mathrm{Tr}\,}}(SP) R \boxtimes W \end{aligned}$$
  which when is traced in $\mathcal {A}_n$ yields
  62 $$\begin{aligned} {{\,\mathrm{Tr}\,}}_{\mathcal {A}_n} \big \{ [(U\boxtimes W) \star ( P\boxtimes Q) ] \star (R\boxtimes S) \big \} ={{\,\mathrm{Tr}\,}}_{N}(QU ) {{\,\mathrm{Tr}\,}}_{N}(RW) {{\,\mathrm{Tr}\,}}_{N}(SP)\,. \end{aligned}$$
  However, if we pick the next observables,
  $$\begin{aligned} O_1&= {\bar{g}}_1 {{\,\mathrm{Tr}\,}}_{N}(UX_a ) {{\,\mathrm{Tr}\,}}_{N}(WX_b )\\ O_2&= {\bar{g}}_2 {{\,\mathrm{Tr}\,}}_{N}(PX_b ) {{\,\mathrm{Tr}\,}}_{N}(QX_c )\\ O_3&= {\bar{g}}_3 {{\,\mathrm{Tr}\,}}_{N}(RX_c ) {{\,\mathrm{Tr}\,}}_{N}(SX_a ) \end{aligned}$$
  the effective vertex for the summand  must be ${{\,\mathrm{Tr}\,}}_{N}(SU ) {{\,\mathrm{Tr}\,}}_{N}(PW) {{\,\mathrm{Tr}\,}}_{N}(QR)$, which is a contradiction with Eq. (62). Thus, Eq. (60a) is impossible. By the same token, one sees that Eq. (60b) leads to an effective vertex ${{\,\mathrm{Tr}\,}}_{N}(PR ) {{\,\mathrm{Tr}\,}}_{N}(QU) {{\,\mathrm{Tr}\,}}_{N}(SW)$, which differs from ${{\,\mathrm{Tr}\,}}_{N}(SU ) {{\,\mathrm{Tr}\,}}_{N}(PW) {{\,\mathrm{Tr}\,}}_{N}(QR)$. Thus, Eq. (60b) is not the right product either.
  To rule out further products, we go to fourth degree in the Hessian. Suppose that Eq. (60e) holds. Then,
  63 $$\begin{aligned}{}[U\boxtimes W \star P\boxtimes Q]\star [ T\boxtimes V \star M\boxtimes L] =&PW\otimes QU \star MV \otimes LT \nonumber \\ =&{\left\{ \begin{array}{ll} PWMV \otimes LTQU \\ MVPW \otimes QULT \end{array}\right. } \end{aligned}$$
  where the last equality lists the possibilities Eq. (54d) or Eq. (54f). In either case, Eq. (60e) holds, then the trace of Eq. (63) reads ${{\,\mathrm{Tr}\,}}_{N}(PWMV) \times {{\,\mathrm{Tr}\,}}_{N}(LTQU)$. Again, if we consider the operators
  64a $$\begin{aligned} O_1&= {\bar{g}}_1 {{\,\mathrm{Tr}\,}}_{N}(UX_a ) {{\,\mathrm{Tr}\,}}_{N}(WX_b )&O_2&= {\bar{g}}_2 {{\,\mathrm{Tr}\,}}_{N}(PX_b ) {{\,\mathrm{Tr}\,}}_{N}(QX_c ) \end{aligned}$$
  64b $$\begin{aligned} O_3&= {\bar{g}}_3 {{\,\mathrm{Tr}\,}}_{N}(TX_c ) {{\,\mathrm{Tr}\,}}_{N}(VX_d )&O_4&= {\bar{g}}_4 {{\,\mathrm{Tr}\,}}_{N}(MX_c ) {{\,\mathrm{Tr}\,}}_{N}(LX_a )\,. \end{aligned}$$
  we get from the summand  in the fourth power of the Hessian the effective vertex ${{\,\mathrm{Tr}\,}}_{N}(LU) {{\,\mathrm{Tr}\,}}_{N}(WP) {{\,\mathrm{Tr}\,}}_{N}(QT) {{\,\mathrm{Tr}\,}}_{N}(VM)$. Since not even the number of traces coincides, Eq. (60e) is impossible. By the same trace-counting argument, the same operators (64) serve as a counterexample for the products Eqs. (60f), (60g) and (60h). This leaves us only with possibilities Eqs. (60c) and (60d):
  65 $$\begin{aligned} (U\boxtimes W) \star ( P\boxtimes Q)&= {\left\{ \begin{array}{ll} Q \boxtimes U {{\,\mathrm{Tr}\,}}_{N}(PW) \\ U\boxtimes Q {{\,\mathrm{Tr}\,}}_{N}(PW) \end{array}\right. } \end{aligned}$$

To finish the proof, we have to determine which of are the correct products, consider the operators

$$\begin{aligned} O_1&={\bar{g}}_1 {{\,\mathrm{Tr}\,}}_{N}(X_a V X_b T ) \,,\\ O_2&={\bar{g}}_2 {{\,\mathrm{Tr}\,}}_{N}(X_b W X_c U ) \,, \\ O_3&={\bar{g}}_3 {{\,\mathrm{Tr}\,}}_{N}(X_cP ) {{\,\mathrm{Tr}\,}}_{N}(X_d Q) \,,\\ O_4&={\bar{g}}_4 {{\,\mathrm{Tr}\,}}_{N}(X_d R ) {{\,\mathrm{Tr}\,}}_{N}( X_a S ) \,, \end{aligned}$$

and the product of their Hessian (entries)

$$\begin{aligned} (T\otimes V \star U\otimes W) \star (P\boxtimes Q \star R \boxtimes S) \end{aligned}$$

This expression is given by

66 $$    \begin{aligned}&= {\left\{ \begin{array}{ll} TU\otimes WV &{} \text {if Eq. } (60c)\text { holds}\\ UT\otimes VW &{} \text {if Eq. } (60d)\text { holds} \end{array}\right. } \Bigg \}\star {\left\{ \begin{array}{ll} S\otimes P {{\,\mathrm{Tr}\,}}_{N}(QR) &{}\quad \text {if Eq. } (54d) \text { holds}\\ P\otimes S{{\,\mathrm{Tr}\,}}_{N}(QR) &{}\quad \text {if Eq. }(54f) \text { holds} \end{array}\right. }\Bigg \} \nonumber \\&= {\left\{ \begin{array}{ll} {{\,\mathrm{Tr}\,}}_{N}(QR) WVSTU\boxtimes P &{}\quad \text {if Eq. } (60c)  \&  \text {Eq. } (54d) \text { hold} \\ {{\,\mathrm{Tr}\,}}_{N}(QR) WVPTU\boxtimes S &{}\quad \text {if Eq. } (60c)  \&  \text {Eq. } (54f) \text { hold} \\ {{\,\mathrm{Tr}\,}}_{N}(QR) VWSUT\boxtimes P &{}\quad \text {if Eq. } (60d)  \&  \text {Eq. } (54d) \text { hold} \\ {{\,\mathrm{Tr}\,}}_{N}(QR) VWPUT\boxtimes S &{}\quad \text {if Eq. }(60d)  \&  \text {Eq. } (54f) \text { hold} \end{array}\right. } \end{aligned}$$

However, since the graphhas an $\mathcal {A}_n$-trace equal to ${{\,\mathrm{Tr}\,}}_{N}(QR) \times {{\,\mathrm{Tr}\,}}_{N}( WPUTSV)$, only the last choice is possible. This proves at once Eqs. (60d) and (54f), that is Eqs. (41d) and (41a), respectively.

### Remark 3

(On well-definedness of the graphical representation.) Example 1 shows a phenomenon that is more general: the asymmetry of the $M_n$-block structure of the nc Hessian matrix, ${{\,\mathrm{Hess}\,}}_{a,b} \ne {{\,\mathrm{Hess}\,}}_{b,a}$. Nevertheless, a weaker symmetry persists. Since the swap of $X_a$ and $X_b$ in Eq. (8) leads to the exchange $\pi _1$ with $\pi _2$, we conclude that for any interaction vertex *O*,

67 $$\begin{aligned} {{\,\mathrm{Hess}\,}}_{a,b} O = \widetilde{{{\,\mathrm{Hess}\,}}_{b,a} O} \quad \text{ where }\,\,\quad \widetilde{(P\otimes Q)}= Q\otimes P\,\,\text{ and }\,\, \widetilde{(P\boxtimes Q)}= Q\boxtimes P\,, \end{aligned}$$

for each $P, Q\in {\mathbb {C}}_{\langle n\rangle }$; the exchange $P\boxtimes Q \rightarrow Q\boxtimes P$ follows by Definition 4. This makes the present construction independent of the choice of “inner” and “outer” loop, as well as the orientation of the interaction vertices in the one-loop (whether the Hessians of $O_1,O_2,\ldots ,O_k$ being multiplied means we draw ${\bar{g}}_1,\ldots ,{\bar{g}}_k$ clockwise or anti-clockwise as in Fig. 2) for the following reason. First, observe that using the algebra obtained in Theorem 2 one can easily derive

68 $$\begin{aligned} \widetilde{{\mathfrak {a}}\star {\mathfrak {b}}} = \tilde{{\mathfrak {b}}} \star \tilde{{\mathfrak {a}} } \qquad \text { for each } {\mathfrak {a}}, {\mathfrak {b}} \in \mathcal {A}_n\,, \end{aligned}$$

Let ${\mathfrak {h}} :={{\,\mathrm{Hess}\,}}_{a_1,a_2} O_1 \star {{\,\mathrm{Hess}\,}}_{a_2,a_3} O_2\star \cdots \star {{\,\mathrm{Hess}\,}}_{a_k,a_1} O_k$, for fixed $a_i=1,\ldots , n$ and for some fixed interaction vertices $O_i$ ($i=1,\ldots ,k$). Then, by Eq. (67)

$$\begin{aligned}&{{{\,\mathrm{Hess}\,}}_{a_1,a_k} O_k} \star \cdots \star { {{\,\mathrm{Hess}\,}}_{a_3,a_2} O_2} \star {{{\,\mathrm{Hess}\,}}_{a_2,a_1} O_1 } \\&= \widetilde{{{\,\mathrm{Hess}\,}}_{a_k,a_1} O_k}\star \widetilde{ {{\,\mathrm{Hess}\,}}_{a_2,a_3} O_2}\star \cdots \star \widetilde{{{\,\mathrm{Hess}\,}}_{a_1,a_2} O_1 } \,, \end{aligned}$$

and because of Eq. (68), this last expression equals $\tilde{{\mathfrak {h}}}$. But according to Lemma 1, ${{\,\mathrm{Tr}\,}}_{\mathcal {A}_n}\tilde{\mathfrak {h}}={{\,\mathrm{Tr}\,}}_{\mathcal {A}_n}\mathfrak {h}$. Thus, if ${\mathfrak {h}}$ contributes to the flow, so does $\tilde{{\mathfrak {h}}}$, and in an equal way, yielding our description independent on the cyclic orientation we choose for drawing the interaction vertices. What we just proved can also be pictorially justified:69Notice that these are the only two representations that the cyclic orientation of each vertex $O_i$ allows (meaning, if one inverts the order of the interaction vertices).

The final piece of well-definedness is that the product found here is indeed associative, without using graphs. The purely algebraic proof is routine (and can be found in [3]).

### Example 6

Once proven the main statement, we can use the algebra to exemplify a typical contribution to the renormalization flow in a Hermitian 3-matrix model. Consider two operators $O_1=\frac{{\bar{g}}_1}{2} [{{\,\mathrm{Tr}\,}}_{N}(\frac{A^2}{2})]^2$ and $O_2 = {\bar{g}}_2 {{\,\mathrm{Tr}\,}}_{N}(ABC)$. Suppose we wish to determine the ${\bar{g}}_1 {\bar{g}}_2^2$-coefficient of the rhs of Wetterich equation. We need (essentially) the Hessian of $O_1$ and $[{{\,\mathrm{Hess}\,}}O_2]^{\star 2}$. The former has only one nonzero entry,70where a “filled ribbon” means that that half-edge is contracted in the one-loop graph, and an “empty ribbon” that it is not (and therefore contributes to the final effective vertex). We also have

71 $$\begin{aligned} {{\,\mathrm{Hess}\,}}O_2= {\bar{g}}_2 \begin{bmatrix} 0 &{}\quad C\otimes 1_N &{}\quad B\otimes 1_N \\ 1_N\otimes C&{}\quad 0 &{}\quad A\otimes 1_N \\ 1_N \otimes B &{}\quad 1_N \otimes A &{}\quad 0 \end{bmatrix} \end{aligned}$$

getting72Only the black-colored entry will contribute, since ${{\,\mathrm{Hess}\,}}O_1$’s (11)-entry is the only non-vanishing. In the (11)-entry, the term(the horizontal green edges still to be contracted in the loop with those in Hessian (70) that are also filled). Finally, we extract the coefficients

$$\begin{aligned}{}[{\bar{g}}_1{\bar{g}}_2]&\mathrm {STr}\{ {{\,\mathrm{Hess}\,}}O_1 [{{\,\mathrm{Hess}\,}}O_2]^{\star 2}\} \\&= {{\,\mathrm{Tr}\,}}_{\mathcal {A}_n}\big \{ [ {{\,\mathrm{Tr}\,}}_{N}(A^2/2) [1_N\otimes 1_N]+A\boxtimes A ] \star [C\otimes C + B\otimes B] \big \} \\&= {{\,\mathrm{Tr}\,}}_{\mathcal {A}_n}\big \{ {{\,\mathrm{Tr}\,}}_{N}(A^2/2) (C\otimes C + B\otimes B ) + A\boxtimes CAC + A\boxtimes BAB \big \} \\&= {{\,\mathrm{Tr}\,}}_{N}(A^2/2) \times [{{\,\mathrm{Tr}\,}}_{N}^2 C +{{\,\mathrm{Tr}\,}}_{N}^2 B ] + {{\,\mathrm{Tr}\,}}_{N}(ACAC+ABAB )\,, \end{aligned}$$

which are effective vertices of the four one-loop graphs that can be formed with the contractions of (the filled ribbon half-edges of)Of course, this is a toy-example: the algebraic structure pays off with higher-power interactions and/or higher number of matrices (whose flow becomes unaccessible by traditional methods and can hardly be cross-checked using graphs, due to the large amount of these; cf. the supplementary material of [3]).

## Conclusion

The algebraic structure of functional renormalization of Hermitian *n*-matrix models with interactions containing several traces has been addressed. Under the assumption that it is possible to compute the flow in terms of $\mathrm U(N)$-invariant operators, the present result completely describes the regulator-independent part of the flow. This paper complements^15^ [3]. There, for multi-matrix models with multi-traces, Wetterich equation was proven, and in the middle of the proof one were able to read off the algebraic structure, (41). Computations of $\beta$-functions using (41) revealed a one-loop structure in [3]. Here we showed the converse: the one-loop structure requires the algebra of functional renormalization (i.e., the structure that makes the rhs of Wetterich equation computable for such matrix models) to be Eq. (41), showing its uniqueness.

As a final perspective, the present results can be useful to connect different renormalization theories, e.g., [30, 31]. Also, Fig. 1 is strikingly reminiscent of the Connes–Kreimer residue defining the coproduct (of their renormalization Hopf-algebra [32]). Between those, the algebraic language could build a shorter bridge than graph theory—all the more, algebra can be coded more directly than graphs.

## Data Availability

Data sharing is not applicable to this article as no datasets were generated or analyzed during the current study.

## Appendix A On $$\Gamma _N$$ and $$ R_N$$

Although our aim in this paper is rather algebraic, we sketch how the FRG works, for sake of completeness. Starting from the bare action $S[\Phi ]$ on $S:\mathcal {H}_N^n \rightarrow {\mathbb {R}}$, in order to construct the effective action $\Gamma _N[{\mathbb {X}}]$ one first regulates the connected partition function $\log {\mathcal {Z}} [{\mathbb {J}}] = \log \int _{\mathcal {H}_N^n} \exp [-S[\Phi ] +\sum _{c=1}^n {{\,\mathrm{Tr}\,}}( J_c \Phi _c)] \mathrm {d}\Phi {}_{\textsc {Leb}}$ (where ${\mathbb {J}}=(J_c)\in \mathcal {H}_N^n$) by adding a mass-like term, $\frac{1}{2} r_{c,N} \Phi ^2_c$, to each matrix:

$$\begin{aligned} {\mathcal {W}}_N [{\mathbb {J}}] = \log \int _{\mathcal {H}_N^n} \exp \bigg \{-S[\Phi ] -\sum _{c=1}^n {{\,\mathrm{Tr}\,}}_{N}\Big [\frac{1}{2} r_{c,N} \Phi ^2_c+ J_c \Phi _a\Big ]\bigg \} \mathrm {d}\Phi {}_{\textsc {Leb}}\end{aligned}$$

where $r_{c,N}$ is the next *infrared regulator*, given in terms of the Heaviside or indicator function $\Theta _{{\mathbb {D}}_N}$ on the disk ${\mathbb {D}}_N=\{ (a,b)\in {\mathbb {N}}^2 \mid a^2+b^2\le N^2\}$ by

A1

with $$Z_c$$ the “wave function renormalization” of the matrix $$X_c$$ (see [3] for details on the dependence on the “classical fields” $${\mathbb {X}}=(X_1,\ldots ,X_n):=(\partial _{J_1} {\mathcal {W}},\ldots ,\partial _{J_n} {\mathcal {W}}) \in \mathcal {H}_N^n $$). Other regulators are not discussed here, but another regulator should comply with the following limits: in order for $$r_{c,N}$$ to integrate out only the “higher modes”, i.e., matrix entries $$\big \{(X_c)_{i,j}\big \}_{i,j\ge N; c=1,\ldots ,n}$$ above the *energy scale* *N*, one imposes that $$r_{c,N}(a,b)=0 $$ if $$a>N$$ or $$b>N$$, which explains the presence of $$\Theta _{{\mathbb {D}}_N}$$ in this particular choice. The additional condition $$r_{c,N}>0$$ creates a mass-like term for the low-energy modes $$\big \{ (X_c)_{i,j}\big \}_{0<i,j< N; c=1,\ldots ,n}$$ that protects these from being integrated out. One wants, moreover, to recover the bare action via saddle point approximation as $$N\rightarrow \infty $$ and thus $$r_{c,N} \rightarrow \infty $$ is necessary in that limit. The interpolating *effective action* is then constructed by taking the Legendre transform, namely $$\Gamma _N[{\mathbb {X}}]= \sup _{J_1,\ldots ,J_n} \sum _{c=1}^n\big \{ {{\,\mathrm{Tr}\,}}_{N}(X_c J_c) -{\mathcal {W}}_N[J_1,\ldots ,J_n] -\frac{1}{2} {{\,\mathrm{Tr}\,}}_{N}(r_{c,N} X_c^2)\big \}$$. The regulator $$R_N$$ that appears in the main text is

$$\begin{aligned} R_N = \begin{bmatrix} r_{1,N} 1_N\otimes 1_N &{} 0&{} \ldots &{} 0 \\ 0 &{} r_{2,N} 1_N\otimes 1_N &{} \ldots &{} 0 \\ \vdots &{} \ddots &{} &{} \vdots \\ 0&{} 0&{} \ldots &{} r_{n,N} 1_N\otimes 1_N \end{bmatrix}. \end{aligned}$$

## Footnotes

1 We should probably write the *functional renormalization group algebra*, but “group algebra” can be confused with ${\mathbb {C}}[ G]$, for a group *G*; or *functional renormalization algebra*, which would suggest, that the renormalization group (which is none) is upgraded to an algebra.

2 These, moreover, might have traces as factors, but being these scalar functions of the matrices that will not be derived again, this is irrelevant.

3 I thank Bertrand Eynard for pointing out the Temperley–Lieb algebra in the context of nc polynomial matrix interactions.

4 This notation has been inspired by Sweedler notation in quantum groups, and avoids to write sums like $Q_{(1)}\otimes Q_{(2)}=\sum _{\alpha } Q_{1,\alpha }\otimes Q_{2,\alpha }$ where each $Q_{1,\alpha },Q_{2,\alpha } \in {\mathbb {C}}_{\langle n\rangle }$.

5 “After” is determined by the scissors pointing in that direction. This is clearer in the graph in Eq. (9).

6 I thank Alexander Schenkel for pointing out that the parameter *t* is still discrete for finite *N* and thus Eq. (14) is not yet a system of differential equations.

7 These graphs are based on the next comment: [24].

8 Notice that the *N* can be re-absorbed in *Z* and the bar-coupling constants, but other conventions are possible.

9 In the single-trace random matrices literature these are sometimes called “stars” [6]. In the definition of the one-dimensional skeleton, it is implicit that we ignore discrete spaces obtained from the many traces that might be floating around the one-dimensional complex. This will be clear in Example 5.

10 The reason for the notation $N_{\infty }$ is that, at the end, that integer can be thought of as being $\infty$. One computes first all with finite $N_{\infty }$ and then takes the limit $N_{\infty }\rightarrow \infty$.

11 This is a historical terminology which should not evoke supersymmetry.

12 Just as the operators ${{\,\mathrm{{\mathscr {D}}}\,}}_A$ and $\partial _A$, this abstract trace is the result of matrix-trace calculations with entries.

13 I thank Răzvan Gurău for questions that motivated this remark (which gives partial answers).

14 Actually one has to trace over $M_n (\mathcal {A}_n)$, i.e., take the supertrace. But the trace corresponding to the $M_n$-block matrix was already taken in Eq. (58).

15 To fully implement the flow for Dirac ensembles, which is the aim of [3] either operators with broken unitary symmetry could be introduced. Another perspective is to implement a Ward-constrained flow [29].
